# Quantitative EEG Insights Into A Hundred Adult ADHD Patients: A Deep Dive Into Test of Variables of Attention (TOVA) Correlations and Attention Dynamics

**DOI:** 10.1111/cns.70304

**Published:** 2025-03-18

**Authors:** Elvan Ciftci, Zeynep Betul Alp

**Affiliations:** ^1^ Department of Psychiatry Uskudar University İstanbul Turkey; ^2^ Department of Neuroscience Uskudar University İstanbul Turkey

**Keywords:** ADHD, ADHD subtypes, automatic linear modeling, delta and high Beta power, qEEG, TOVA

## Abstract

**Objective:**

This study aimed to enhance the diagnostic accuracy of attention‐deficit/hyperactivity disorder (ADHD) by integrating quantitative electroencephalography (qEEG) power bands with the test of variables of attention (TOVA) and self‐reported psychiatric symptoms. We examined the relationship between TOVA scores, qEEG findings—particularly the theta‐beta ratio—and comorbid psychiatric conditions to assess their role in refining ADHD diagnoses according to the Diagnostic and Statistical Manual of Mental Disorders, Fifth Edition (DSM‐V).

**Method:**

A total of 100 participants were assessed using TOVA, qEEG, and psychological scales, including the Beck Anxiety Inventory (BAI), Beck Depression Inventory (BDI), Maudsley Obsessive‐Compulsive Inventory (MOCI), and the Mood Disorder Questionnaire (MDQ). Participants were categorized into groups based on their Attention Comparison Scores (ACS) above or below the zero threshold. Mann–Whitney *U*‐tests, correlation analyses, and predictive modeling using automatic linear modeling (ALM) were conducted to evaluate group differences, age‐related changes, and predictor variables for attention performance.

**Results:**

All participants met the diagnostic criteria for ADHD. Among them, 37% exhibited anxiety, 60% depression, 26% obsessive‐compulsive, and 35% mood disorder symptoms. The group with ACS above zero was significantly older (*p* = 0.034) and performed better on all Tests of Variables of Attention (TOVA) measures (*p* < 0.05). Age negatively correlated with attention scores (*r* = −0.371, *p* < 0.001), response time variability (*r* = −0.241, *p* = 0.016), and response time (*r* = −0.311, *p* = 0.002). qEEG showed significant age‐related changes in theta‐to‐beta and delta‐to‐beta ratios (*p* < 0.005). TOVA and qEEG ratios, particularly beta and delta activity, predicted attention and response time variability, with adjusted *R* ^2^ values between 71.5% and 87.1%.

**Conclusion:**

The findings highlight that attention performance in ADHD is shaped by age, neuropsychological factors, and qEEG‐measured brain activity. Higher attention scores correlate with better TOVA results, particularly in response time and error rates. Age‐related declines in attention align with reductions in theta‐to‐beta and delta‐to‐beta ratios. Predictive modeling underscores the value of combining TOVA and qEEG to identify key predictors like response time variability, omission errors, and specific beta and delta activity. This integration enhances the evaluation of attention deficits and brain dynamics, benefiting both clinical and research applications.

## Graphical abstract

This graphical abstract synthesizes a comprehensive ADHD study, showcasing how combining qEEG and TOVA metrics with demographic data through ALM can enhance diagnostic accuracy, particularly emphasizing the significant inverse relationship between age and theta/beta ratios in ADHD symptomatology.

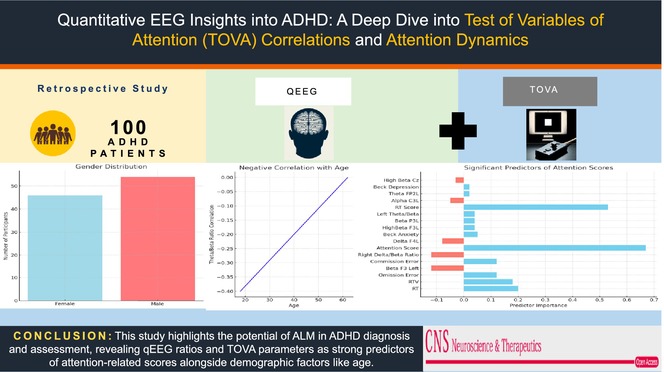

## 1 Introduction

Attention‐deficit hyperactivity disorder (ADHD) is a neurodevelopmental condition affecting approximately 2.5%–6.7% of the global adult population and 5%–7.2% of children [1, 2]. Historically, the DSM‐IV categorized ADHD into three subtypes based on dominant symptomatology: inattentive, hyperactive–impulsive, or combined [3]. As outlined by the American Psychiatric Association's DSM‐V, children under 17 are diagnosed with ADHD if they exhibit six or more symptoms from the inattentive, hyperactive–impulsive domains, or both. In contrast, adults need to show at least five such symptoms [4]. The DSM‐V updated the age threshold for symptom onset from 7 years, as previously defined in DSM‐IV, to 12 years, broadening the scope for adult diagnosis. To reflect the evolving nature of symptom manifestations as patients' age, DSM‐V replaced the term “subtype” with “presentation.” Notably, while 25% of adults with ADHD are primarily inattentive, 70% fit the combined presentation criteria. The hyperactive–impulsive presentation is rare, appearing in < 5% of adult ADHD cases [5].

ADHD is severe and frequently diagnosed in childhood; despite its prevalence, ADHD remains notably underdiagnosed and inadequately treated among adults [6]. ADHD is more common in boys than in girls in children and adolescents, with ratios ranging from 3:1 to 10:1, but in adults, the distribution of prevalence across genders is more balanced [7]. According to population cohort studies, the adult population with ADHD may not only consist of people whose symptoms began in childhood and continued into adulthood, but also of at least 20% of late‐onset cases that started in late adolescence or early adulthood [8] which contradicts the traditional neurodevelopmental theory of ADHD [9]. The clinical presentation in adulthood evolves, resulting in frequent persistent disabilities: hyperactivity is more frequently internalized, and symptoms of inattention may be masked by anxiety symptoms or obsessive‐like compensatory strategies. In adults, other symptoms such as emotional deregulation or executive function‐related symptoms are common [10]. Clinical monitoring has demonstrated that ADHD can fluctuate or disappear at any point in life, including in adulthood [11].

ADHD is frequently associated with other psychiatric disorders in adults (in 80% of cases), including personality disorders, especially borderline and antisocial, substance use disorders (SUD), anxiety disorders, and mood disorders [12] and making diagnoses even more difficult. Addressing ADHD promptly and effectively can potentially modify the trajectory of subsequent psychiatric conditions and considerably improve outcomes related to associated psychiatric comorbidities.

A delay in development and cortical hypo‐activation due to a lack of dopaminergic and noradrenergic system function has been associated with central nervous system dysfunction, which has been linked to ADHD [13]. Low cortical activation associated with the dopaminergic and noradrenergic systems may account, at least partially, for the inhibitory and attentional deficits that characterize ADHD [14]. Innovative analytical techniques treating EEG as a three‐dimensional functional imaging tool, through its waveform patterns, are providing deeper insights into the neurophysiological nuances. The distinct pattern of electro‐cortical activity at rest has been seen in ADHD subjects. Less activation in central and central‐prefrontal areas is typically associated with inattentive presentations, whereas hyperactive/impulsive presentations are linked to poor activation in left prefrontal areas. Pre‐frontal (e.g., Fp1, Fp2, Fp3) and central (e.g., Cz) regions have been the most frequently reported areas for ADHD [15]. Specifically, slow waveform activities correlate with decreased blood flow and reduced energy (glucose) consumption in the corresponding brain region, which in turn reflects the arousal state of the ADHD subject [16]. Numerous studies have observed that individuals with ADHD typically exhibit diminished alpha and beta wave activities, but heightened delta and theta wave activities [17]. Notably, a prominent theta/beta ratio disparity exists when comparing ADHD subjects to neurotypical controls [16].

In light of these challenges, employing precise diagnostic tools is crucial for accurate identification and subsequent management of ADHD. Among the commonly utilized diagnostic tools, the Test of Variables of Attention (TOVA) holds significance not only in diagnosing ADHD but also in monitoring treatment efficacy. This continuous performance test has been rigorously evaluated and standardized for both pediatric and adult populations [18].

In this study, we explored the relationships between quantitative electroencephalography (qEEG) variables and TOVA performance metrics, in addition to anxiety, depression, mood disorder, and obsessive‐compulsive symptom ratings in patients with ADHD. It is crucial to clarify that qEEG variables are not directly measured by the TOVA test; instead, we analyzed how these EEG patterns correlate with TOVA scores, which assess cognitive functions such as attention, reaction time, and impulsivity. We also examined whether comorbid psychiatric disorders influence qEEG patterns, potentially affecting scores on these TOVA parameters.

This investigation allowed us to test the hypothesis that qEEG patterns are modified in the presence of comorbid psychiatric disorders in individuals with ADHD, impacting key cognitive parameters measured by TOVA, including Attention Comparison Score (ACS), reaction time (RT), reaction time variability (RTV), commission errors (CE), and omission errors (OE).

## 2 Method

### 2.1 Study Design and Participants

This retrospective study was conducted on subjects with a primary diagnosis of ADHD based on the Diagnostic and Statistical Manual of Mental Disorders (DSM‐V) criteria aged 18–62 years from an outpatient psychiatry hospital in Turkey. From a database of 215 patients with ADHD and comorbid psychiatric disorders, 100 patients (46 female, 54 male) aged 28.5 + −15.75 median ± interquartile range (IQR) (minimum (min)–maximum (max)) were retrospectively selected based on our specific criteria. Our selection criteria considered the patients' prominent symptoms and existing diagnoses. This process excluded patients with other primary psychiatric diagnoses such as substance abuse or psychosis.

The ADHD diagnoses of the included patients were validated by a psychiatrist and a psychologist following psychiatric evaluations. During the evaluation process, results from qEEG, TOVA, and rating scales were taken into account. It is important to note that comorbid psychiatric diagnoses were also clinically established by licensed psychiatrists in accordance with standard diagnostic criteria. The scales utilized in the study, including [insert specific scales], were exclusively employed for assessing and grading symptom severity, rather than serving as standalone diagnostic tools. This ensured that clinical diagnoses were determined independently, with the scales providing supplementary quantitative data to support the evaluations.

While these tools provide valuable insights into symptom severity and cognitive performance, the extent to which qEEG and TOVA results alone can predict an ADHD diagnosis according to standard diagnostic procedures remains unclear. The focus of this study is to enhance the accuracy of ADHD diagnoses by improving existing diagnostic methods and acknowledging the limitations of using qEEG and TOVA as standalone diagnostic tools.

Ethical approval for the study procedures was obtained from the Ethics Committee of a Uskudar university in Turkey (dated: May 31, 2023, approval no: 61351342/May 2023–2023).

### 2.2 Data Collection

Data were collected from medical records, including demographic information, clinical diagnoses, and results from the TOVA, qEEG, and standardized psychological assessment tools.

### 2.3 TOVA

To support the diagnosis of ADHD and determine its severity, various objective diagnostic techniques have been developed. The TOVA is a specialized, culture‐ and language‐free computerized test designed to assess attention deficits, including ADHD. It records responses to visual or auditory stimuli using a highly accurate microswitch, eliminating the need for left/right discrimination or sequencing. The test consists of four quarters and takes approximately 20 min to complete. During the GO/NO‐GO task, participants are required to press a button in response to “GO” stimuli, represented by squares appearing on the screen, while refraining from responding to “NO‐GO” stimuli. Stimuli are displayed on a 19‐in. screen, with patients seated 1.5 m away for optimal visibility [19].

### 2.4 TOVA Captures Behavioral Metrics

#### 2.4.1 The ACS

The ACS in the TOVA test is a measure used to compare an individual's performance against the normative data of individuals diagnosed with ADHD. This score is calculated by considering three main variables: the response time from the first half of the test, the performance measure from the second half, and the total variability observed throughout the test. These metrics assess how long and effectively an individual can maintain their attention.

#### 2.4.2 Response Time

This measures how quickly an individual responds during the test. It reflects the ability to respond accurately and swiftly to targets. This is the average speed of correct responses to targets, providing a measure of information processing speed. Motor response speed and an increase in response time can be considered indicators of depression. If there is a slowdown in this parameter alone throughout the test, it may be interpreted as psychomotor slowing, suggesting the possibility of obsessive‐compulsive disorder (OCD) or a depressive process.

#### 2.4.3 Response Time Variability (RTV)

This shows how consistent an individual's response times are; high variability may indicate fluctuations in attention. This measures the variations in correct response times, reflecting the consistency or inconsistency in responses. This is referred to as “micro attention,” focusing on the attention span between the 2 s between stimuli to see if a person “drifts” when attending to the visual or auditory test. RTV, which is calculated as the standard deviation of RT, is thought to be a measure of consistency or variability of attention as an indicator for obsessive behaviors, and the total score (T‐SS) [19] is calculated. An increase in RTV in the first quartile indicates performance anxiety.

#### 2.4.4 Omissions Errors (OE)

This likely refers to the number of correct responses missed, reflecting focus and vigilance and measuring inattention.

#### 2.4.5 Commissions (CE)

This likely refers to the number of incorrect responses to non‐target stimuli and a measure of response inhibition or impulsivity.

#### 2.4.6 Performance Measure

Commonly referred to as D Prime, this value measures how well an individual can distinguish between correct hits (targets) and false alarms (non‐targets).

These values are divided by a calibration constant. The calibration constant is used to standardize test results and make comparisons possible between different individuals or groups. For example, if the response time is −1.57, the performance measure is −1.00, total variability is −1.86, and the calibration constant is 1.80, the ACS would be calculated as −2.62.

An ACS value below zero indicates that the individual's performance is closer to that of the ADHD normative sample, suggesting a more likely ADHD profile. This score is used as an indicator of an individual's attention and concentration abilities and is evaluated based on specific attention characteristics.

While the ACS obtained from the TOVA test ranges between −14 and +14, it's crucial to understand that a positive score does not definitively indicate the absence of ADHD. For instance, individuals with a score such as +2.40 might still exhibit atypical values in response time variability, response time, commission errors, or omission errors. Therefore, although a score above zero is generally seen as favorable, it should not be considered conclusive on its own. Each individual's results must be evaluated in a broader context, taking into account a combination of various test measures. It is important to emphasize that TOVA test results serve merely as one tool and should be interpreted alongside other clinical findings and observations to make a comprehensive ADHD diagnosis. This approach helps to clarify the complex nature of the ACS and highlights that it cannot be used as a standalone diagnostic tool.

A primary challenge in assessing ADHD effectively is the variation in symptom presentation, influenced by the patient's age and gender. The TOVA compares these measurements to a large age‐ and gender‐matched normative sample, as well as to a sample population of individuals independently diagnosed with ADHD. These comparisons are utilized to create an immediately available, easy‐to‐read report. These scores are displayed in the comparison to the normative sample and in the ACS, which help provide an understanding of the subject's performance in both low arousal and high arousal conditions.

The TOVA's approach is consistent with the broader understanding of ADHD assessment, which emphasizes the importance of a nuanced evaluation of attention‐related variables.

In summary, the TOVA facilitates a structured, objective assessment of attention and inhibitory control variables crucial for ADHD evaluation, thus aiding clinicians in understanding and diagnosing ADHD, alongside other diagnostic methods.

### 2.5 Psychological Assessment Instruments

Participants completed a suite of self‐report measures, which included the following.

#### 2.5.1 Beck Anxiety Inventory (BAI)

A 21‐item scale measuring anxiety severity with a total score range of 0–63. Anxiety classifications: low: < 18, moderate: < 25, high: ≥ 25. (BAI > 18 is accepted as anxiety threshold).

#### 2.5.2 Beck Depression Inventory (BDI)

Evaluates depression severity. Turkish cut‐off scores: mild: 0–12, moderate: 13–18, severe: 29–63. (BDI > 17 is accepted as depression threshold).

#### 2.5.3 Maudsley Obsessive Compulsive (OC) Inventory (MOCI) [20, 21]

Assesses obsessive‐compulsive symptoms. OCD likelihood: Low: ≤ 12, Possible: 13–17, High: ≥ 18. (MOCI > 18 is accepted as OC threshold).

#### 2.5.4 Mood Disorder (MD) Questionnaire (MDQ) [22, 23]

Measures lifetime manic or hypomanic symptoms with a cut‐off score of 7 indicating likely mood disorders. (MDQ > 7 is accepted as MD threshold).

#### 2.5.5 DSM‐IV‐Attention Deficit and Hyperactivity Disorder Scale [24, 25]

A 23‐item measure based on DSM‐IV criteria for adult ADHD. Symptom presence is indicated by a score of 2 or higher. (DSM‐ADHD > 20 is accepted as the ADHD threshold).

All assessments were administered in their validated Turkish translations.

### 2.6 qEEG Procedure

In our study, the qEEG data collection was conducted in a controlled environment to minimize distractions and ensure reliable recordings. Participants underwent an 8.5‐min EEG recording session involving a series of structured activities. These included initial closed‐eye recording, alternating eye movements, hyperventilation, photic stimulation, and concluding with a final closed‐eye period. Electrode placement adhered to the 10–20 international system with 21 electrodes, using linked mastoids (M1–M2) as reference points.

The EEG data were sampled at 500 Hz, filtered to remove noise, and artifacts were manually excluded post‐collection. The analysis focused on the absolute power of various brain waves (Delta, Theta, Alpha, Beta, and High Beta) across different scalp regions. This comprehensive qEEG approach was designed to investigate the neurophysiological correlates of attention deficiency and hyperactivity/impulsivity in ADHD.

### 2.7 Statistical Analysis

All statistical calculations were performed using IBM SPSS Statistics for Windows version 22.0 [26].

The data did not follow a normal distribution, as indicated by the Kolmogorov–Smirnov test (*p* < 0.05). Detailed results are provided in Table S1. Therefore, non‐parametric tests were applied. The Mann–Whitney *U*‐test was used to compare two independent groups, and Spearman's correlation was employed for correlation analyses. Descriptive results were presented as mean and standard deviation for normally distributed variables, and median with interquartile range for non‐normally distributed variables. A *p* value of 0.05 was considered statistically significant.

### 2.8 Automatic Linear Modeling

Automatic linear modeling (ALM) was utilized for its machine learning capabilities in simplifying the model selection process within IBM SPSS 22.0. The ALM automatically scales input variables, such as sex, age, qEEG parameters, and psychological assessment scores, to facilitate analysis. Using a training set of one hundred patient records, the model followed the F Statistic criterion for a step‐by‐step construction. The process efficiently identified significant predictors influencing the TOVA scores by assessing the weighted contributions of variables like age, sex, and various qEEG ratios. ALM's automated variable selection highlighted its value in predictive modeling within this study.

## 3 Results

Using TOVA as an assessment tool, 79 participants (79%) showed indications that may suggest ADHD. The BAI assessment pointed to possible anxiety symptoms in 37 individuals (37%). According to the BDI evaluation, 60 participants (60%) exhibited signs that could indicate depression. MOCI results suggested the presence of OCD symptoms in 26 participants (26%). The MDQ assessment indicated potential mood disorders in 35 individuals (35%). Finally, all the patients fulfilled the criteria of DSM‐ADHD criteria; 100 participants (100%) showed signs that may be consistent with ADHD.

### 3.1 Comparison of TOVA Parameters, Clinical Variables, and qEEG Between Above and Below ACS Indices

We compared the ADHD patient sample with scores above and below the ACS zero threshold by using the Mann–Whitney *U*‐test. The ACS is a composite metric derived from TOVA parameters, compared against a normative ADHD sample based on age and sex. For the purposes of this study, the continuous ACS scale was converted into a dichotomous index (above or below zero) to facilitate group comparisons. The group having ACS above zero did not differ significantly in terms of the distribution of sexes from the group with scores below zero (*p* > 0.05). Age (above/below ACS: median + −IQR (min‐max) = 30 ± 15 (18–62) / 23 ± 7.5 (18–43)) of the above ACS group was statistically higher than that of the below ACS group (*p* < 0.05). All of the TOVA parameters, attention score, RTV, RT, CE, or OE were all significantly different between the two groups (*p* < 0.05) (Table 1). All the other clinical variables, including the psychological test score results and all qEEG variables, were not significantly different from each other between the two groups (*p* < 0.05). When we compared all qEEG parameters and ratios, we only found a significant difference for high beta F3 left between the two groups (*p* = 0.047).

**TABLE 1:** TOVA and clinical variables.

Mann–Whitney *U* (mean rank)	Sex	Age	Attention score	RT variability	RT	Commission error	Omission error
Above ACS (*n* = 21)	6F, 15M	53.66	78.88	73.86	64.45	79.02	79.79
Below ACS (*n* = 79)	40F, 39M	38.62	42.96	44.29	46.79	42.92	42.92
*Z*	−1.794	−2.114	−5.044	−4.159	−2.481	−5.079	−5.275
Asymp. Sig. (2‐tailed)	0.073	**0.034**	** < 0.001**	** < 0.001**	**0.013**	** < 0.001**	** < 0.001**

Abbreviation: IQR: Interquartile Range is given. *p* < 0.05 is the significance level.

### 3.2 Correlation of Age With TOVA Parameters and qEEG Ratios

In our statistical analysis of TOVA performance metrics and age, we uncovered significant correlations. Specifically, the attention score showed a negative correlation with age, as evidenced by Spearman's rho (*r* = −0.371, *p* < 0.001). RTV and RT also negatively correlated with age (*r* = −0.241, *p* = 0.016 for RTV and *r* = −0.311, *p* = 0.002 for RT). These results imply that as age increases, certain attention‐related abilities may decline. Contrarily, the relationships between age and both CE and OE were not significant (*p* > 0.05), indicating these particular aspects of attention were less affected by age. Additionally, qEEG ratios revealed significant age‐related changes. A significant negative correlation was observed between age and certain qEEG ratios, as shown in Table 2, suggesting alterations in brain activity patterns with age (*p* < 0.005).

**TABLE 2:** Correlation of age with TOVA parameters and qEEG ratios.

Spearman's rho	ACS	RTV	RT	Theta to high beta left	Theta to high beta right	Theta to high beta central	Theta to beta right	Theta to beta central	Theta to beta all	Theta to alpha left	Delta to beta central	Delta to beta right	Delta to beta central
Age	−0.317 (0.001)	−0.241 (0.016)	−0.311 (0.002)	−0.201 (0.045)	−0.390 (< 0.001)	−0.367 (< 0.001)	−0.344 (< 0.001)	−0.346 (< 0.001)	−0.354 (< 0.001)	0.243 (0.015)	−0.282 (0.004)	−0.262 (0.008)	−0.282 (0.004)

*Note:* Correlation coefficients (*p* value significance level) are given.

Abbreviations: ACS, Attention Comparison Score; RT, response time; RTV, response time variability.

### 3.3 Correlation Between Comorbid Psychiatric Disorder Scores and qEEG Ratios

When we evaluated the correlation between the scores or presence of comorbid psychiatric disorders and qEEG ratios, we found correlations to be significant at the 0.05 level (two‐tailed) (Table 3).

**TABLE 3:** Correlation between comorbid psychiatric disorder scores and qEEG ratios.

Spearman's rho	BAI score	Anxiety presence	BDI Score	Depression presence	MOCD Score	Mood disorder presence	ADHD presence
Theta to high beta left				0.219 (0.029)			
Theta to high beta right	0.281 (0.019)			0.264 (0.008)	0.199 (0.047)	0.252 (0.034)	0.259 (0.031)
Theta to high beta central		0.268 (0.026)	0.220 (0.028)				
Theta to beta right			0.214 (0.032)				
Theta to beta central			0.257 (0.010)				
Theta to beta all			0.253 (0.011)				
Theta to alpha right		0.326 (0.006)		0.312 (0.009)			
Theta to alpha central		0.350 (0.003)		0.301 (0.012)			

*Note:* Correlation coefficients (*p* value significance level) are given.

Abbreviations: ADHD, attention deficiency and hyperactivity; BAI, Beck Anxiety Inventory; BDI, Beck Depression Inventory; MOCD, Maudsley Obsessive Compulsive Disorder.

Significant correlations between comorbid psychiatric disorder scores, QEEG ratios and TOVA parameters results are given in the Table 4

**TABLE 4:** Correlation between comorbid psychiatric disorder scores, qEEG ratios, and TOVA parameters.

Spearman's rho	BAI score	BDI score	MOCD score	MDQ score	DSM‐IV ADHD score	ACS	RTV	RT	CE	OE	High beta Pz	Theta Pz
BAI		0.577 *p* < 0.001	0.482 *p* < 0.001		0.584 *p* < 0.001							
BDI	0.577 *p* < 0.001	0.482 *p* < 0.001	0.431 *p* < 0.001		0.594 *p* < 0.001							
MOCD	0.482 *p* < 0.001	0.431 *p* < 0.001		0.272 *p* = 0.023	0.393 *p* = 0.001							
MDQ			0.272 *p* = 0.023		0.421 *p* < 0.001	−0.264 *p* = 0.026	−0.280 *p* = 0.018					0.239 *p* = 0.044
DSM‐IV ADHD	0.584 *p* < 0.001	0.594 *p* < 0.001	0.393 *p* = 0.001	0.421 *p* < 0.001		−0.348 *p* = 0.003	−0.390 *p* = 0.001	−0.382 *p* = 0.001				
ACS				−0.264 *p* = 0.026	−0.348 *p* = 0.003		0.906 *p* < 0.001	0.750 *p* < 0.001	0.330 *p* = 0.001	0.782 *p* < 0.001		
RTV				−0.280 *p* = 0.018	−0.390 *p* < 0.001	0.906 *p* < 0.001		0.789 *p* < 0.001		0.642 *p* < 0.001		
RT					−0.382 *p* = 0.001	0.750 *p* < 0.001	0.789 *p* < 0.001			0.488 *p* < 0.001		
CE						0.330 *p* = 0.001				0.377 *p* < 0.001		
OE						0.782 *p* < 0.001	0.642 *p* < 0.001	0.488 *p* < 0.001	0.377 *p* < 0.001		0.199 *p* = 0.047	

Abbreviations: ACS, Attention Comparison Score; BAI, Beck Anxiety Inventory; BDI, Beck Depression Inventory; CE, commissions; DSM‐IV ADHD, DSM‐IV attention deficiency and hyperactivity; MDQ, Mood Disorder Questionnaire; MOCD, Maudsley Obsessive Compulsive Disorder; OE, omissions errors; RT, response time; RTV, response time variability.

### 3.4 Modeling Indices via ALM

#### 3.4.1 ACS Modeling

**FIGURE 1:**
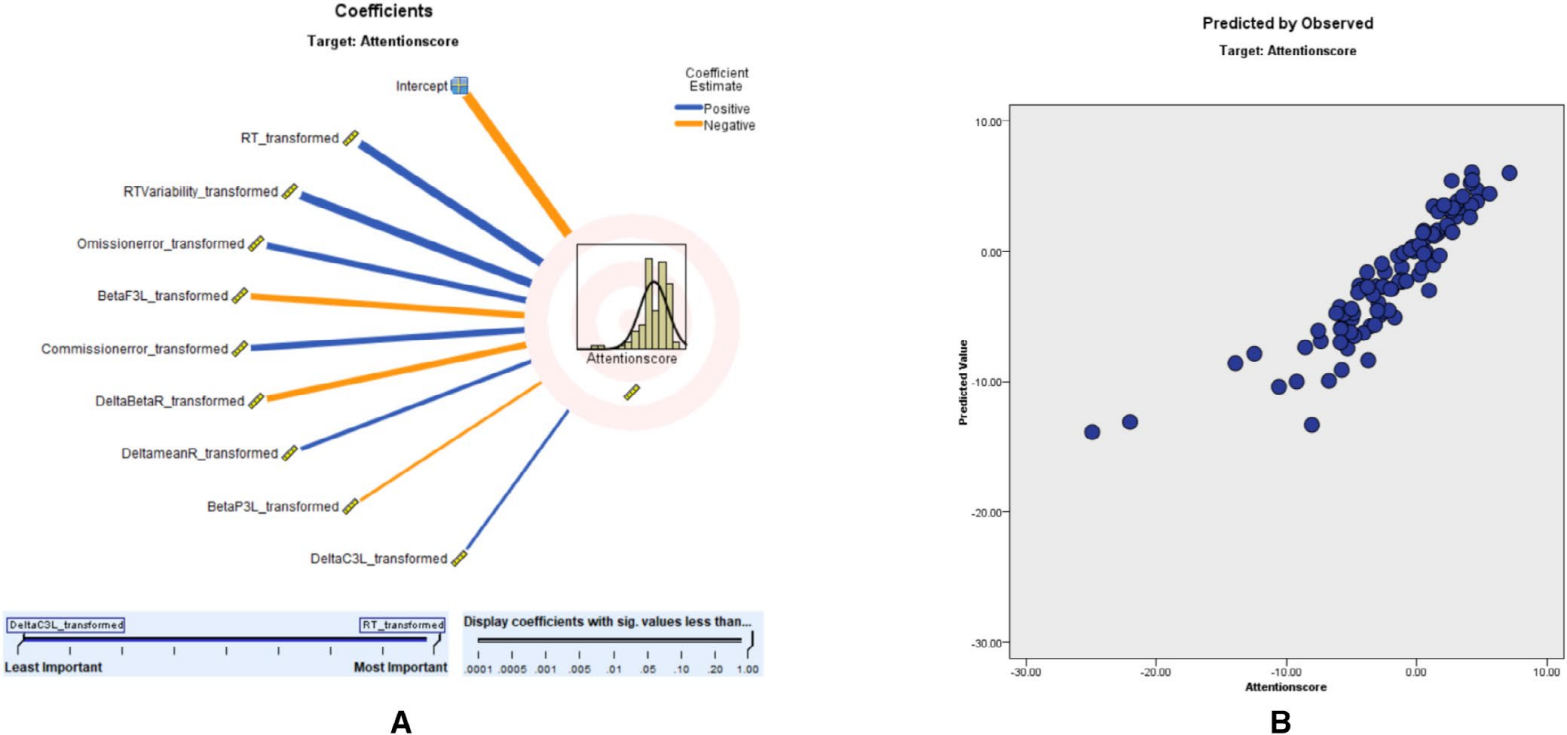
Attention score modeling: (A) The correlation strength and direction of various predictors on attention scores. Blue lines indicate positive correlations, while orange lines represent negative correlations. (B) predicted versus observed attention scores, demonstrating the model's accuracy. Data points clustering along the 45° line reflects the model's strong predictive reliability.

In the predictive modeling conducted via ALM, we utilized the ACS as our primary dependent variable to measure attention parameters. This decision was made due to the ACS being the most informative and relevant scale we had at our disposal for evaluating attentional aspects in the context of TOVA. The ACS served as a robust metric, correlating with various predictors, including qEEG variables, demographic factors, and comorbid psychological conditions. Our model yielded an adjusted *R* ^2^ value of 79.6%, reflecting a strong fit. Moreover, the scatter plot analysis reinforced the model's consistency, with a substantial proportion of data points aligning with the 45° line, indicative of a reliable predictive relationship. The predictor importance (coefficient value) shows that RT (0.20, positively correlated), RTV (0.18, positively correlated), omission error (0.12, positively correlated), beta F3 left (0.12, negatively correlated), commission error (0.12, positively correlated), right delta‐to‐beta ratio (0.12, negatively correlated), right delta mean value (0.06, negatively correlated), left beta P3 (0.04, positively correlated), and left delta C3 (0.04, negatively correlated) correlates with attention score These findings, presented in Figure 1, affirm the ACS's validity in reflecting attention scores within our study. Further details of the ACS prediction model, including predictor importance, residual distributions, outlier analysis, and model‐building summaries, are provided in Tables S2–S6.

#### 3.4.2 RTV Modeling

**FIGURE 2:**
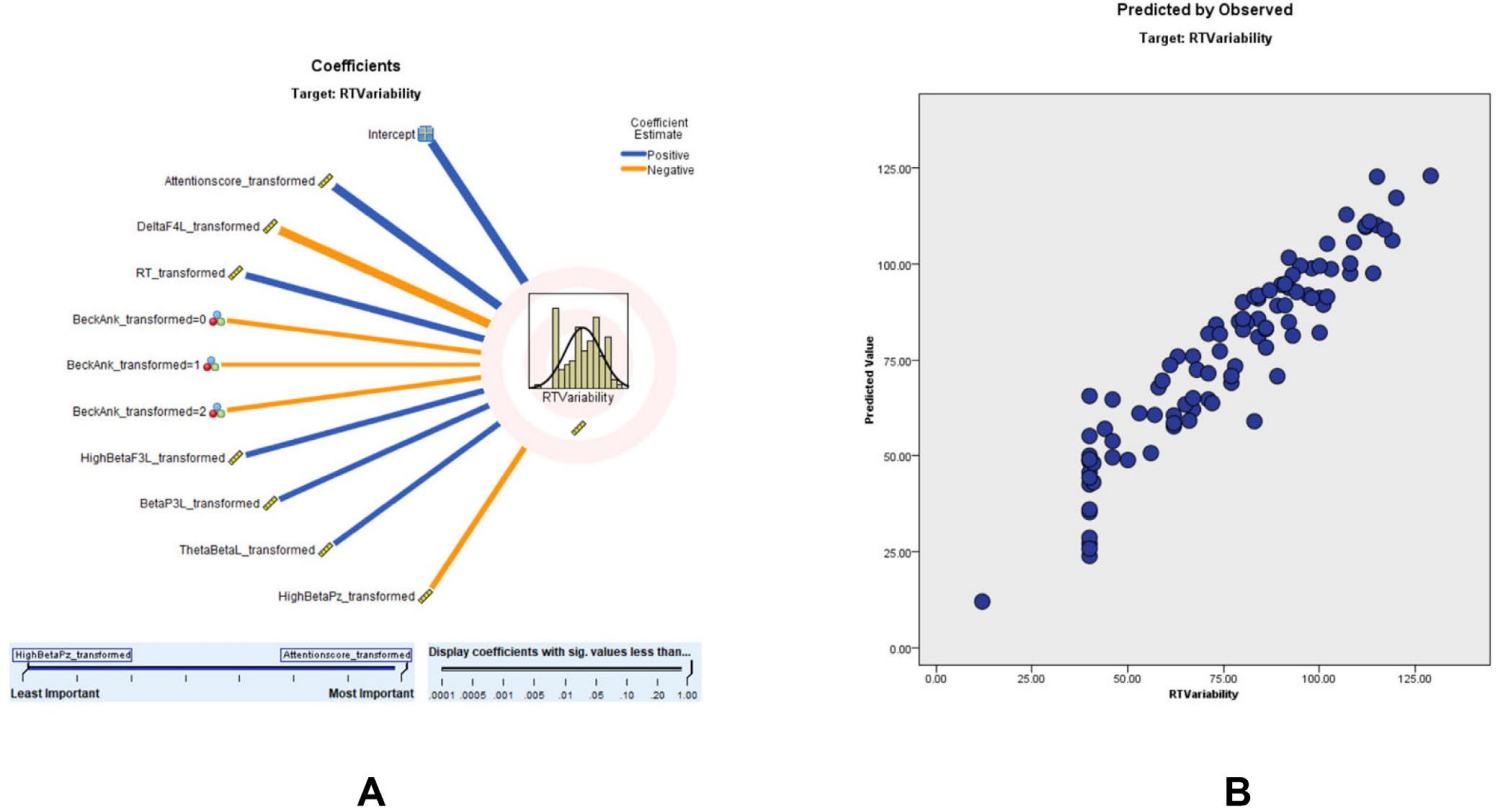
RTV modeling: (A) displays the influence of various predictors on response time variability (RTV). Predictors with the strongest positive contributions are highlighted in blue, while those with negative associations are marked in orange. This visualization helps identify key factors affecting RTV. (B) compares the predicted RTV values to the observed values, reflecting the consistency and accuracy of the model. The alignment of data points along the diagonal suggests robust predictive performance.

Modeling for RTV revealed an adjusted *R* ^2^ of 87.1%, with the ACS being the strongest predictor. According to the RTV scatter plot, a higher percentage of the sampling points fit on the 45° line, and the model is relatively consistent. The predictor importance (coefficient value) shows that ACS (0.67, positively correlated), delta F4 left (0.08, negatively correlated), RT (0.05, positively correlated), beck anxiety (0.05), high beta F3 left transformed (0.04, positively correlated), beta P3 left (0.04, positively correlated), left theta‐to‐beta ratio (0.04, positively correlated) and high beta PZ (0.04, negatively correlated) correlate with RTV (Figure 2). Detailed results of the RTV prediction model, including predictor importance, residual distributions, outlier analysis, and model‐building summaries, can be found in Tables S7–S11.

#### 3.4.3 RT Modeling

**FIGURE 3:**
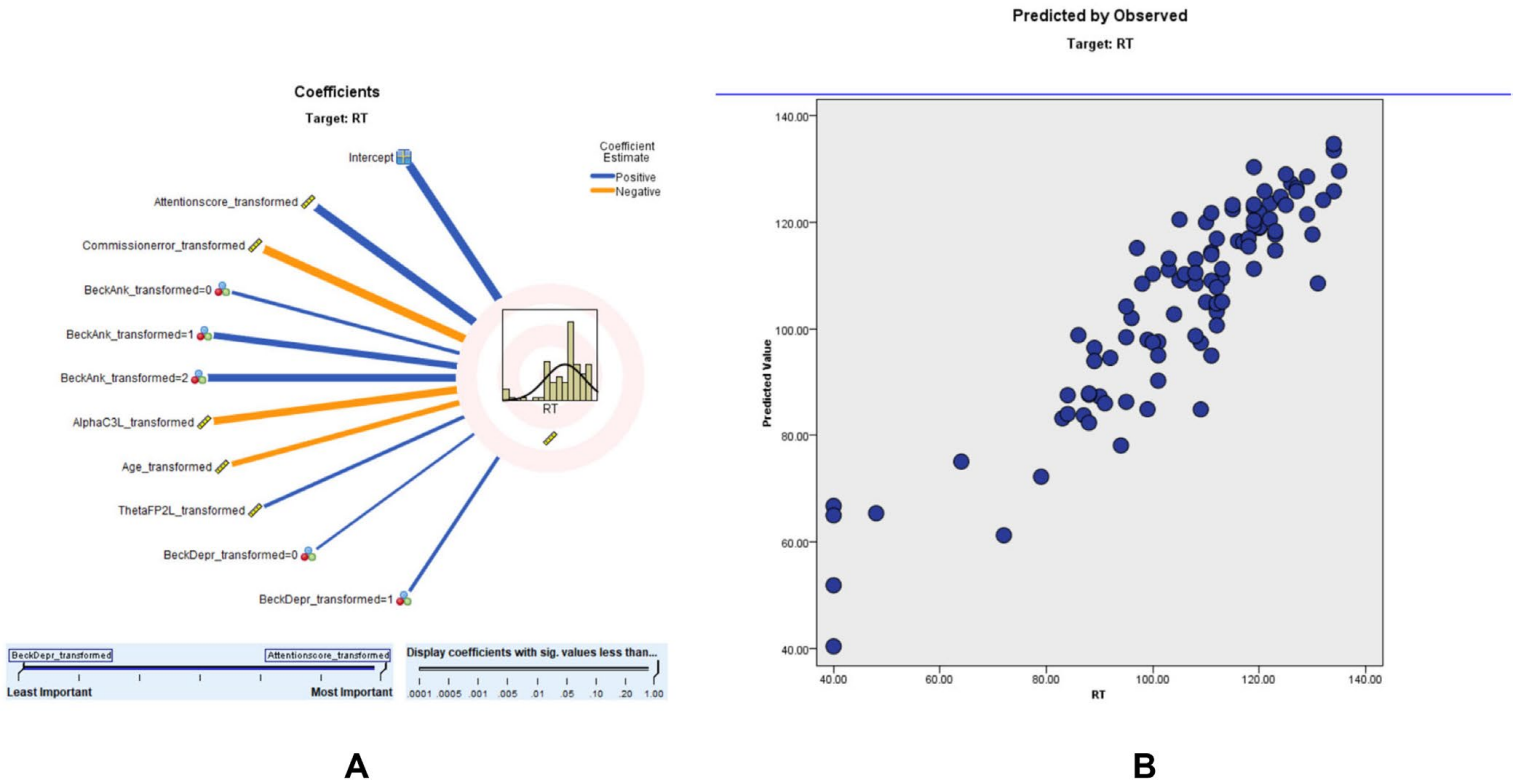
RT Modeling: (A) illustrates the correlation strength and direction of various predictors on response time (RT). Predictors with positive contributions are represented by blue lines, while negative correlations are shown in orange. (B) depicts the predicted versus observed RT values, indicating the model's performance. The clustering of data points along the 45° line reflects the predictive accuracy and reliability of the model.

Similar modeling for RT showed an adjusted *R* ^2^ of 80.7%, with ACS again being a significant predictor. According to the RT scatter plot, a higher percentage of the sampling points fit on the 45° line, and the model is relatively consistent. The predictor importance (coefficient value) shows that RT score (0.53, positively correlated), commission error score (0.29, negatively correlated), beck anxiety (0.06), alpha C3 left (0.05, negatively correlated), age (0.03, negatively correlated), theta FP2 left (0.02, positively correlated), and beck depression (0.02) correlate with RT (Figure 3). Comprehensive results of the RT prediction model, including predictor importance, residual distributions, outlier analysis, and model‐building summaries, are provided in Tables S12–S16.

#### 3.4.4 CE Modeling

**FIGURE 4:**
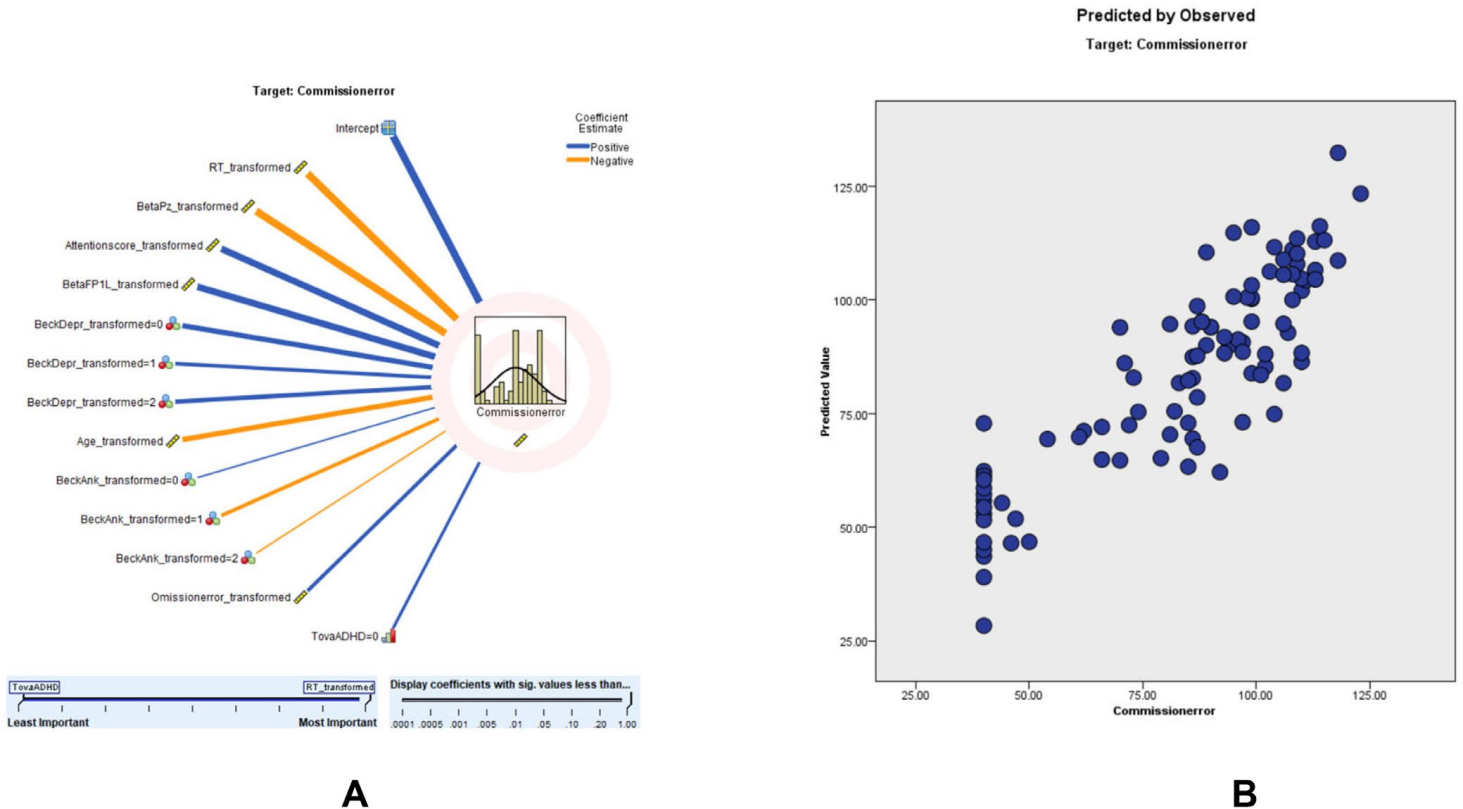
Commission Error Modeling: (A) illustrates the correlation strength and direction of various predictors on commission errors. Blue lines indicate positive correlations, while orange lines represent negative correlations. (B) displays the predicted versus observed commission errors, showing the model's accuracy. The alignment of data points along the diagonal suggests the model's reliability in predicting commission errors.

Commission error modeling had an adjusted *R* ^2^ of 71.5%, with RT being the strongest predictor. According to the CE scatter plot, a higher percentage of the sampling points fit on the parallel line, and the model is relatively consistent. The coefficient value shows that RT (0.38, negatively correlated), beta Pz (0.14, negatively correlated), attention score (0.11, negatively correlated), beta FP1 left (0.11, positively correlated), beck depression (0.09), age (0.06, negatively correlated), beck anxiety (0.05), omission error (0.03, positively correlated), and TOVA supporting ADHD (0.02, negatively correlated) correlate with CE (Figure 4). Detailed findings of the commission error (CE) prediction model, including predictor importance, residual distributions, outlier analysis, and model‐building summaries, are presented in Tables S17–S217.

#### 3.4.5 OE Modeling

**FIGURE 5:**
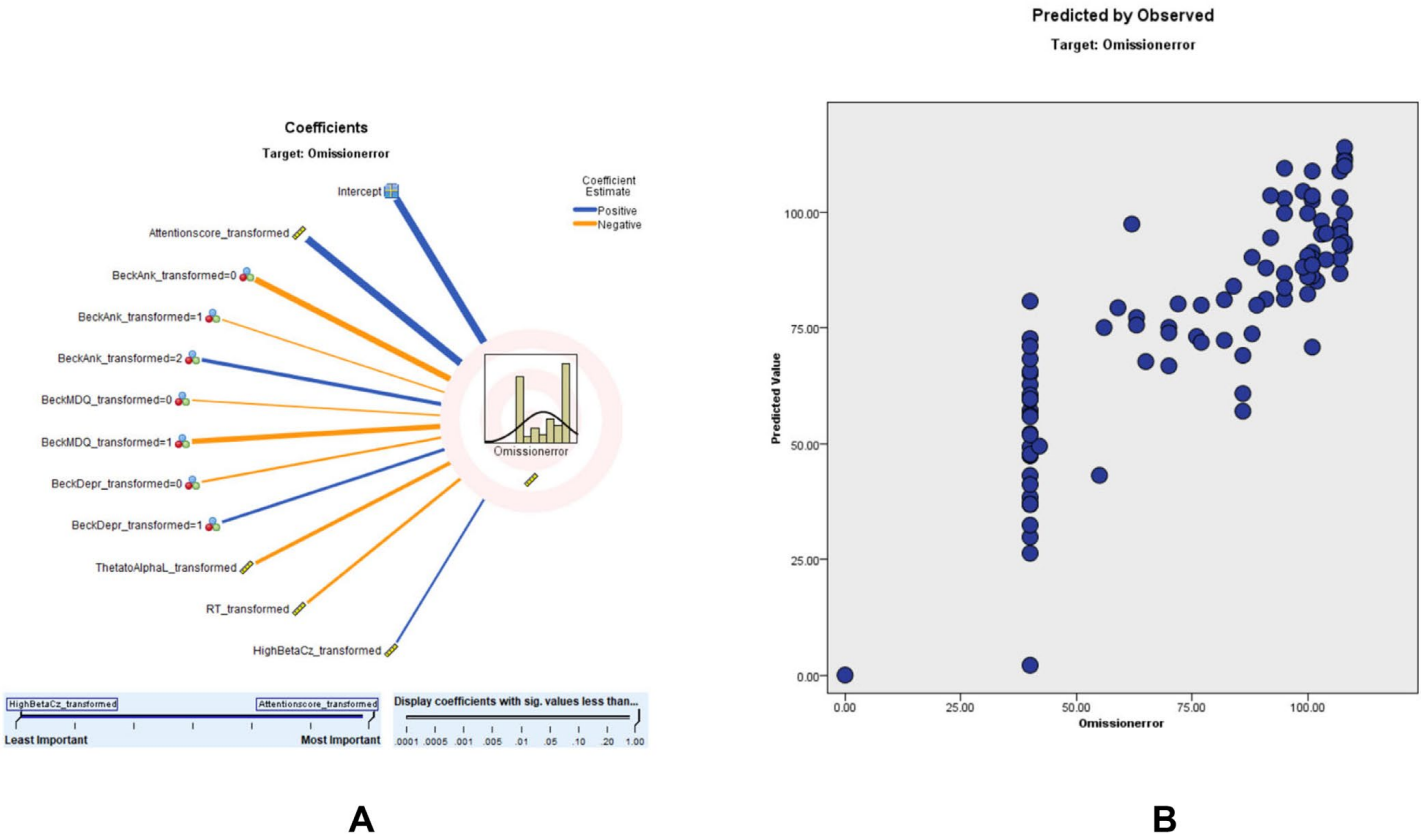
Omission error modeling: (A) shows the correlation strength and direction of various predictors on omission errors. Blue lines represent positive correlations, while orange lines indicate negative correlations. (B) illustrates the predicted versus observed omission errors, highlighting the model's accuracy. The clustering of data points along the diagonal reflects the reliability of the model in predicting omission errors.

OE modeling showed an adjusted *R* ^2^ of 69.1%, with the DSM‐ADHD transformed score as the top predictor. OE score. According to the OE scatter plot, a higher percentage of the sampling points fit on the 45° and 90° lines, and the model is relatively consistent. The coefficient value shows that ACS (0.51, positively correlated), beck anxiety score (0.20), Mood Disorder Questionnaire (MDQ, 0.11), Beck depression (0.06), theta‐to‐alpha left (0.05, positively correlated), RT (0.03, positively correlated), and high Beta Cz (0.03, negatively correlated) correlate with OE (Figure 5).

These findings illustrate that while age is a significant factor in TOVA performance, qEEG ratios and specific TOVA parameters are powerful predictors of attention‐related scores in ADHD assessments. Comprehensive results of the omission error (OE) prediction model, including predictor importance, residual distributions, outlier analysis, and model‐building summaries, are provided in Tables S22–S26.

## 4 Discussion

### 4.1 Age With Tova Parameters

The findings from the Results section underscore the impact of age on attentional capacities, aligning with prior studies that have also noted the decline of cognitive functions with age. The lack of a significant correlation between commission and omission errors with age suggests that these errors might be influenced by other factors not captured in this study. The observed decrease in qEEG theta to beta or high beta power ratio (but not only theta power) with increased age could be indicative of normal aging processes affecting neural efficiency, which in turn influences attention parameters.

We would like to highlight that our study found no significant correlation between the theta‐to‐beta ratio and ACS. Additionally, although the theta‐to‐beta or high‐beta ratio decreases with age, we observed that attention scores also decline with age. This suggests that age significantly impacts attention performance in our participants, beyond the symptoms of ADHD.

The significant yet weak correlations between age and TOVA scores suggest that while age is a contributing factor to attentional performance, it is not the sole determinant. This highlights the complex nature of attentional capacities and the potential for other variables, such as neurocognitive development or compensatory mechanisms, to play roles in attentional outcomes in older adults.

Moreover, the adjusted *R* ^2^ value of 79.6% for our model, which uses qEEG and demographic factors to predict the TOVA ACS, demonstrates a strong model fit. This suggests that our predictors effectively explain variations in attention scores and merit further investigation for clinical use.

### 4.2 Integration With qEEG Studies

The primary goal of our study was to clarify the associations between qEEG power bands, TOVA performance, and psychological assessment metrics in individuals with ADHD. Consistent with previous research, such as that by Konopka [27], our results highlight the capability of qEEG to reflect the neurophysiological aspects of ADHD, particularly through the significant correlation between theta power and inattention scores. This reaffirms the value of qEEG in identifying EEG patterns linked with attentional and response control deficits characteristic of ADHD.

In the adult population, ADHD rarely occurs in isolation, often presenting alongside other psychiatric conditions, which complicates the diagnostic process. Aligning with findings by Katzman et al. [6], our data reveal a high incidence of comorbid psychiatric disorders in patients seeking treatment for ADHD, with notable percentages for depression, anxiety, mood disorders, and OCD. This underscores the need for a comprehensive assessment strategy that considers the broad spectrum of psychiatric comorbidities prevalent among individuals with ADHD.

The intricate interplay of ADHD and its comorbid conditions necessitates a tiered approach to treatment, prioritizing the most severe disorder, as also advocated by Katzman et al. [6]. They suggest that early intervention may improve long‐term psychiatric outcomes, advocating for the use of validated diagnostic tools and targeted clinical interviews to identify adults with ADHD and offer them evidence‐based treatments.

Our analysis further supports the combined application of TOVA and qEEG as a more definitive method for assessing attentional deficits and inhibitory control. The correlation between qEEG signals and TOVA scores underscores the potential for these tools to evolve into more sophisticated and objective diagnostic modalities for ADHD.

The TOVA assessment in our study revealed significant differences in attention scores, RTV, CE, and OE between individuals with scores above and below the ACS threshold. This delineation is in line with existing research, such as the meta‐analysis by Kofler et al. [28], which discusses the increased RTV in ADHD and its diagnostic relevance, and studies by Tamm et al. [29] and Epstein et al. [30], which emphasize RTV's prominence in cognitive research relating to ADHD.

Additionally, the negative correlation between age and the theta/beta ratio, along with the delta/beta and theta/high beta ratios in the central region, suggests an age‐related modulation in EEG activity. This trend extends to the TOVA parameters of attention score, RT, and RTV, hinting at more pronounced symptoms in younger individuals and providing insight into the evolving nature of ADHD across different age groups. The relationship between qEEG metrics and ADHD symptoms highlights the utility of resting‐state EEG as a reliable marker for ADHD, paving the way for more nuanced and age‐sensitive diagnostic criteria.

### 4.3 Machine Learning (ML), Automatic Linear Modeling (ALM) and TOVA

ALM sits at the intersection of statistical analysis and ML, offering innovative approaches to psychiatric diagnosis, as exemplified by recent advances in EEG applications. Park et al. [31] demonstrated the viability of ML in distinguishing psychiatric disorders from resting‐state EEG data. Our study contributes to this burgeoning field by examining the interplay between qEEG patterns and TOVA scores, reinforcing the potential of EEG‐based ML models in clinical diagnostics.

Our use of ALM, as introduced by Yang [32] in the LINEAR procedure of SPSS 21.0, was instrumental in managing a multitude of variables, streamlining the analysis of demographic and psychological assessment data. This efficient variable selection, crucial in identifying predictors such as attention score and response time, reduced manual input and expedited our study's analytical phase.

Cowley et al. [33] highlighted the role of theta and alpha oscillations in attentional processing in ADHD, findings that resonate with our own. In our study, ALM revealed predictors such as left beta F3, left beta P3, left delta C3, right delta mean value, and right delta/beta ratio as significant for attention scores. These results suggest that differential patterns in slow and fast qEEG oscillations have predictive value for ADHD, a notion supported by Rodríguez et al. [34] who found a stronger relationship between activation in central and left prefrontal areas and task execution in ADHD subjects.

Our study expands on these insights, showing the dynamic nature of ADHD across age groups, reflected in the significant negative correlations between age and certain qEEG ratios. The ADHD symptomatology appears to be modulated by both age and neural oscillatory patterns, with implications for diagnosis and treatment strategies.

The attentional dynamics in ADHD are multifaceted. Abbass et al. [35] demonstrated the relevance of the theta band and the frontoparietal alpha band in processing visual targets, insights that are echoed by our findings on the predictive value of left alpha C3, left theta FP2 for RT, and theta/alpha left, high beta Cz for OE. These electrophysiological markers, along with those identified by Ogrim et al. [36], underscore the intricate ADHD landscape, where both neurophysiological and behavioral analyses are crucial for a comprehensive understanding.

In summary, our research underlines the importance of left frontal beta (F3), left parietal beta (P3), left central delta (C3), right delta mean value, and right hemisphere delta/beta ratio as qEEG‐based predictors for ADHD attention scores, suggesting potential regional brain anomalies in ADHD symptom manifestation. These patterns warrant further investigation to elucidate the complex neurophysiological mechanisms underlying ADHD.

### 4.4 Anxiety, ADHD, and qEEG

qEEG measurements in patients with anxiety disorders have revealed distinct patterns, such as increased beta waves [37] and alpha wave asymmetry [38]. These patterns are attributed to heightened frontal cortex activity and variations in cortical arousal [39]. When considering ADHD, particularly with comorbid anxiety, these qEEG signatures become even more significant.

In our study, anxiety was a notable predictor of TOVA parameters like RTV, RT, OE, and CE, echoing Jang et al.'s [40] findings on the influence of comorbid anxiety on qEEG patterns in ADHD children. This is particularly relevant in understanding TOVA's commission errors, which may reflect a blend of impulsivity and anxiety‐driven responses.

Moreover, Ribas et al. [37] explored anxiety‐specific qEEG patterns that could intersect or interact with ADHD patterns. This interaction is crucial when analyzing reaction time variability or omission errors in TOVA. Our findings regarding attention with a correlation between increased delta power in the central region and beta power in the left parietal region (P3), and decreased executive function is in line with Bong et al. [41]. In the context of anxiety and executive functioning, the absolute delta power seems to play a specific role in the task‐negative default mode network, highlighting the complex interplay between anxiety and ADHD symptomatology.

### 4.5 Depression, Bipolar Disorder, ADHD, and qEEG

The significance of emotional dysregulation in adults with ADHD is defined by the study of Richard‐Lepouriel et al. [42], which also reveals that these individuals exhibit higher emotional intensity compared to controls and subjects with bipolar disorder. Even though the current diagnostic criteria for ADHD do not include an emotional dimension, the care for emotional responsiveness in patients with the inattentive and hyperactive/impulsive components needs to be improved.

Mood disorders, including depression and bipolar disorder (BD), often share symptomatology with ADHD. In our study, we found that depression was a significant predictor of RT, CE, and OE in ADHD, while mood disorder overall predicted omission error. This aligns with findings from Kim et al. [43], who observed a correlation between left and right frontal alpha powers in major depressive disorder (MDD) and the Adult ADHD Self‐Report Scale (ASRS) scores, both for inattention and the total score.

In cases of BD, Kim et al. [43] noted a significant association of right frontal gamma power with ASRS hyperactivity and total scores. Their linear regression analyses further revealed that, in MDD, ASRS inattention and total scores correlated significantly with frontal alpha powers, depression, and lifetime hypomania episodes. Conversely, in BD, ADHD‐related hyperactivity was linked to lifetime hypomania and right frontal gamma power.

These findings elucidate that frontal alpha power in MDD patients is associated with ADHD symptoms, whereas in BD, right frontal gamma power correlates with ADHD symptomatology. This underscores the complex neurological underpinnings shared between mood disorders and ADHD, as reflected in qEEG patterns, and highlights the need for nuanced diagnostic approaches that consider these overlapping features.

### 4.6 OCD, ADHD, and qEEG

The interplay between ADHD and OCD has been a subject of debate for over two decades, with inconsistent co‐occurrence rates reported in the literature. While many studies suggest a comorbidity between ADHD and OCD, substantial etiological (genetic) support is predominantly found in pediatric populations. It has been proposed that the increased rates of ADHD‐OCD co‐occurrence might be mediated by tic disorders. Additionally, impaired neuronal maturation processes observed in pediatric OCD could lead to ADHD‐like symptoms, posing diagnostic challenges as these may be mistaken for primary ADHD symptoms [44].

In line with these findings, our research also faces challenges related to the complexity of disorder severity and comorbidity. These factors underscore the necessity for more nuanced interpretations in future research. A limitation, as also identified by Abramovitch et al. [44], is the need for broader and more diverse sample validation to enhance the generalizability of findings.

The increasing use of EEG and ML in psychiatric research heralds a new era of more individualized and evidence‐based approaches to diagnosis and treatment. In this context, our study contributes to the understanding of ADHD and OCD, emphasizing the importance of discerning overlapping symptomatology for accurate diagnosis. As EEG technology and ML algorithms continue to evolve, they hold the potential to untangle the intricate relationship between these disorders, thereby aiding in the development of more targeted and effective therapeutic interventions.

### 4.7 Limitations

Nevertheless, there are certain limitations intrinsic to our study. Firstly, the modest sample size could inhibit a detailed stratification based on ADHD subtypes, which may affect the generalizability of our findings. It would be prudent for future research to encompass a more expansive dataset to shed light on potential variations across different ADHD subtypes. Secondly, the lack of a longitudinal design restricts our understanding of the temporal evolution of EEG patterns and their relationship with attentional dynamics over time. Furthermore, the demographic variability within our sample was not thoroughly explored, which could limit the applicability of our findings across diverse populations. Potential subject selection bias and the adequacy of control group comparisons might have also impacted the robustness of our results.

Another limitation of this study is that, in our initial analysis, we utilized the Automatic Linear Modeling (ALM) method, which optimizes model fit by selecting significant variables but does not inherently adjust for the risk of Type I errors associated with multiple comparisons. Future analyses will address this by incorporating such corrections to ensure the robustness of our findings, thereby mitigating the risk of spurious results due to multiple testing.

Finally, a significant limitation of this study is the occasional mismatch between ADHD symptoms observed in daily life and the outcomes of standard laboratory tests, especially evident in continuous performance tests like TOVA. This discrepancy highlights the complexity of ADHD diagnosis and reflects the unique coping mechanisms patients employ in daily life, underscoring the need for diversification in diagnostic methodologies. This suggests that future research should embrace more comprehensive approaches.

## 5 Conclusion

Our study highlighted the significance of employing narrow diagnostic categories to enhance classification accuracy, particularly in the context of ADHD. By delving into the relationship between specific qEEG signatures and TOVA scores, we have illuminated a potential pathway for refining diagnostic precision. Our findings suggest that associating distinct qEEG patterns with TOVA scores could pave the way for identifying EEG markers unique to varying ADHD presentations. This, in turn, holds promise for fostering a more precise diagnostic and treatment framework, not only for ADHD but also for understanding comorbid psychiatric disorders. The insights garnered from our study suggest further exploration in larger and more diverse cohorts to validate and extend our understanding. Moreover, integrating a multidisciplinary approach, encompassing behavioral, neurophysiological, and perhaps genetic analyses, could propel us closer to a more nuanced and effective diagnostic paradigm for ADHD and its comorbidities.

## Conflicts of Interest

The authors declare no conflicts of interest.

## Supporting information


Appendix S1.

## Data Availability

The data supporting the findings of this study are available from the corresponding author upon reasonable request. Due to privacy and ethical restrictions, the data are not publicly available.
