# Supplementary material for: Quantitative EEG Insights Into A Hundred Adult ADHD Patients: A Deep Dive Into Test of Variables of Attention (TOVA) Correlations and Attention Dynamics
Source: CNS Neurosci Ther. 2025 Mar 18;31(3):e70304. doi: 10.1111/cns.70304 (PMC11919765; doi:10.1111/cns.70304)
Supplement: Supplementary file 1 — Appendix S1. [file CNS-31-e70304-s001.docx]

**Supplementary Table S1: Normality Test of Variables**

|  | Kolmogorov-Smirnov^a^ | | |
| --- | --- | --- | --- |
|  | Statistic | df | Sig. |
| TovaADHD | .472 | 67 | .000 |
| Sex | .351 | 67 | .000 |
| Age | .156 | 67 | .000 |
| Attentionscore | .062 | 67 | .200^*^ |
| RTVariability | .080 | 67 | .200^*^ |
| RT | .135 | 67 | .004 |
| Commissionerror | .164 | 67 | .000 |
| Omissionerror | .172 | 67 | .000 |
| DeltaFP1L | .136 | 67 | .004 |
| DeltaF3L | .494 | 67 | .000 |
| DeltaC3L | .114 | 67 | .031 |
| DeltaP3L | .458 | 67 | .000 |
| DeltameanL | .424 | 67 | .000 |
| ThetaFP1L | .168 | 67 | .000 |
| ThetaF3L | .180 | 67 | .000 |
| ThetaC3L | .186 | 67 | .000 |
| ThetaP3L | .209 | 67 | .000 |
| ThetameanL | .132 | 67 | .006 |
| AlphaFP1 | .136 | 67 | .004 |
| AlphaF3L | .126 | 67 | .010 |
| AlphaC3L | .136 | 67 | .004 |
| AlphaP3L | .194 | 67 | .000 |
| AlphameanL | .135 | 67 | .004 |
| BetaFP1L | .118 | 67 | .021 |
| BetaF3L | .107 | 67 | .054 |
| BetaC3L | .151 | 67 | .001 |
| BetaP3L | .145 | 67 | .001 |
| BetameanL | .120 | 67 | .017 |
| HighBetaFP1L | .204 | 67 | .000 |
| HighBetaF3L | .150 | 67 | .001 |
| HighBetaC3L | .151 | 67 | .001 |
| HighBetaP3L | .262 | 67 | .000 |
| HighBetameanL | .160 | 67 | .000 |
| DeltaFP2L | .179 | 67 | .000 |
| DeltaF4L | .132 | 67 | .006 |
| DeltaC4L | .168 | 67 | .000 |
| DeltaP4L | .194 | 67 | .000 |
| DeltameanR | .131 | 67 | .006 |
| ThetaFP2L | .144 | 67 | .001 |
| ThetaF4L | .178 | 67 | .000 |
| ThetaC4L | .450 | 67 | .000 |
| ThetaP4L | .227 | 67 | .000 |
| ThetameanR | .342 | 67 | .000 |
| AlphaFP2L | .133 | 67 | .005 |
| AlphaF4L | .124 | 67 | .013 |
| AlphaC4L | .490 | 67 | .000 |
| AlphaP4L | .170 | 67 | .000 |
| AlphameanR | .382 | 67 | .000 |
| BetaFP2L | .110 | 67 | .044 |
| BetaF4L | .122 | 67 | .015 |
| BetaC4L | .146 | 67 | .001 |
| BetaP4L | .167 | 67 | .000 |
| BetameanR | .137 | 67 | .003 |
| HighBetaFP2L | .194 | 67 | .000 |
| HighBetaF4L | .127 | 67 | .009 |
| HighBetaC4L | .432 | 67 | .000 |
| HighBetaP4L | .425 | 67 | .000 |
| HighBetameanR | .292 | 67 | .000 |
| DeltaFz | .124 | 67 | .013 |
| DeltaCz | .175 | 67 | .000 |
| DeltaPz | .160 | 67 | .000 |
| DeltameanC | .129 | 67 | .007 |
| ThetaFz | .167 | 67 | .000 |
| ThetaCz | .189 | 67 | .000 |
| ThetaPz | .235 | 67 | .000 |
| ThetameanC | .189 | 67 | .000 |
| AlphaFz | .122 | 67 | .015 |
| AlphaCz | .122 | 67 | .014 |
| AlphaPz | .148 | 67 | .001 |
| AlphameanC | .126 | 67 | .010 |
| BetaFz | .142 | 67 | .002 |
| BetaCz | .145 | 67 | .001 |
| BetaPz | .143 | 67 | .002 |
| BetameanC | .147 | 67 | .001 |
| HighBetaFz | .419 | 67 | .000 |
| HighBetaCz | .478 | 67 | .000 |
| HighBetaPz | .464 | 67 | .000 |
| HighBetameanC | .409 | 67 | .000 |
| Anx | .390 | 67 | .000 |
| BeckAnk | .116 | 67 | .025 |
| Depr | .382 | 67 | .000 |
| BeckDepr | .119 | 67 | .019 |
| OCD | .413 | 67 | .000 |
| MaudsleyOCD | .101 | 67 | .086 |
| MDQ | .382 | 67 | .000 |
| BeckMDQ | .140 | 67 | .002 |
| DSMADHD | .100 | 67 | .093 |
| ThetaHighBetaL | .174 | 67 | .000 |
| ThetaBetaL | .104 | 67 | .067 |
| ThetaHighBetaR | .400 | 67 | .000 |
| ThetaBetaR | .345 | 67 | .000 |
| ThetaHighBetaC | .182 | 67 | .000 |
| ThetaBetaC | .172 | 67 | .000 |
| DeltaBetaL | .438 | 67 | .000 |
| DeltaBetaR | .123 | 67 | .013 |
| DeltaBetaC | .093 | 67 | .200^*^ |
| ThetatoBetaAll | .220 | 67 | .000 |
| ThetatoAlphaL | .296 | 67 | .000 |
| ThetatoAlphaR | .376 | 67 | .000 |
| ThetatoAlphaC | .188 | 67 | .000 |

Results of the Kolmogorov-Smirnov normality test for TOVA parameters, QEEG metrics, and clinical variables. Variables with p < 0.05 indicate a significant deviation from normal distribution, suggesting the need for non-parametric statistical methods.

**ALM Analysis**

**Supplementary Table S2: ACS Prediction**


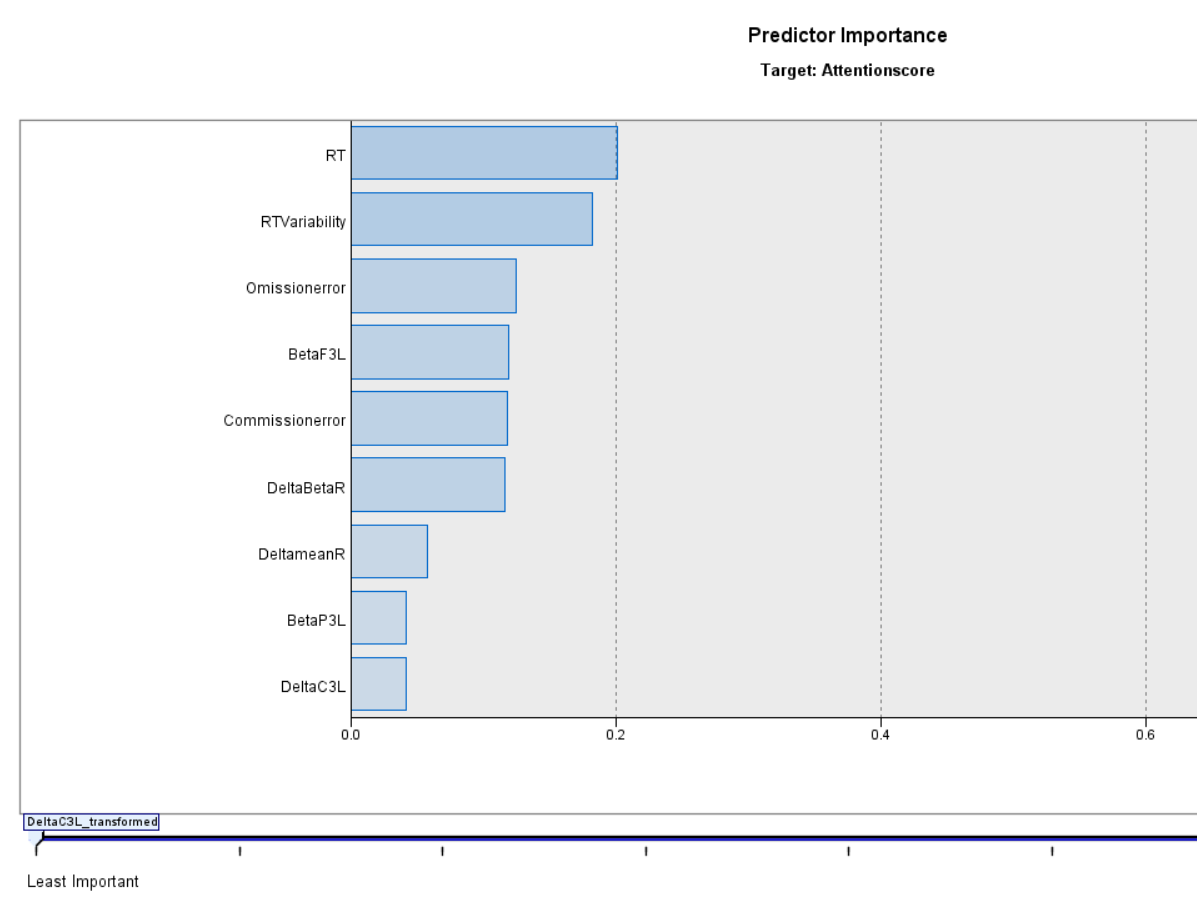


Predictor importance values for Attention Control Score (ACS) prediction, showing the relative contribution of each variable to the model. Higher values indicate greater importance in predicting ACS.

**Supplementary Table S3: Residual Distribution for ACS Prediction**


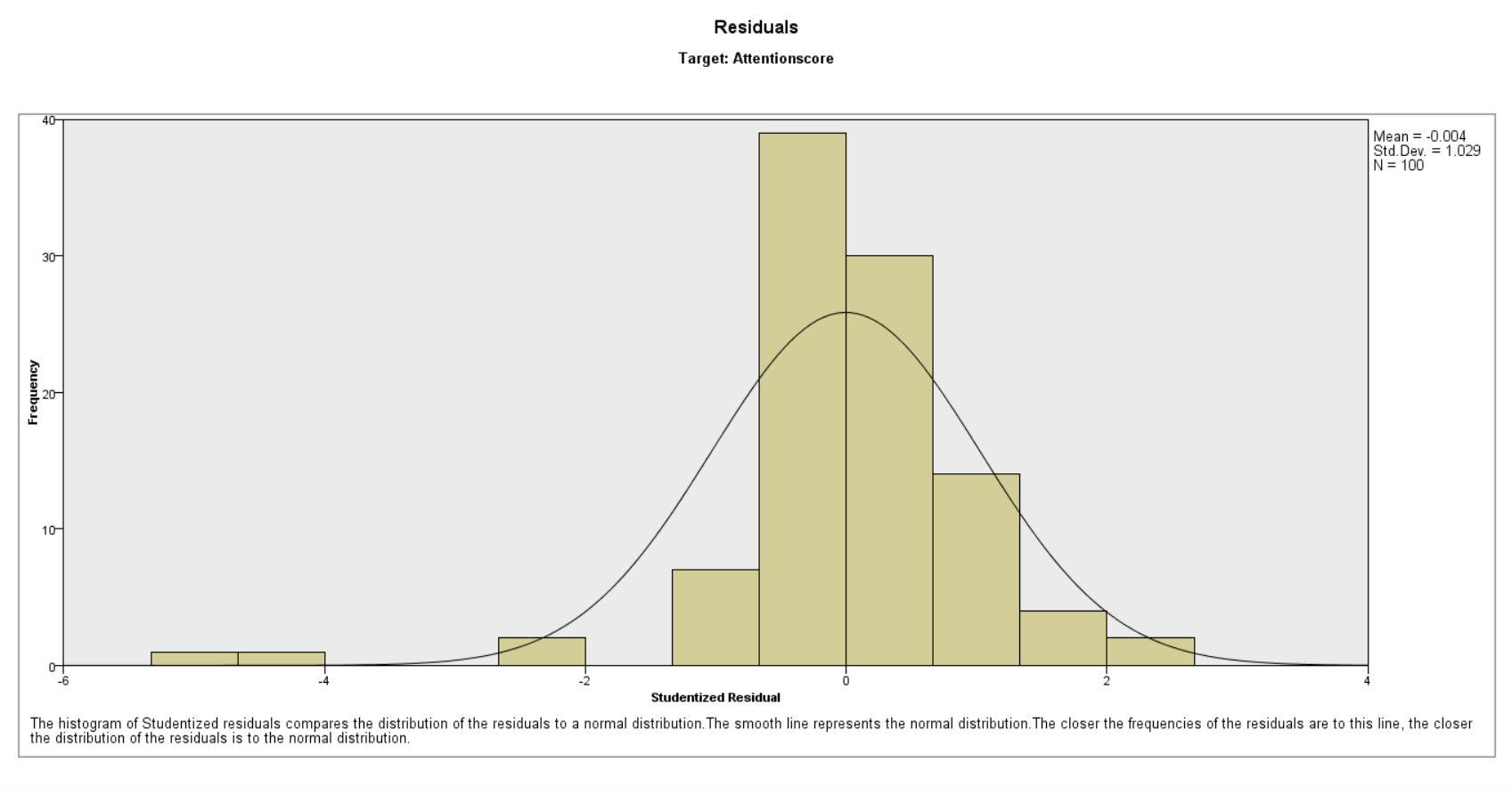


The histogram displays the distribution of studentized residuals for the Attention Control Score (ACS) prediction model. The smooth line represents the normal distribution. The closer the residual frequencies align with the line, the better the residuals fit a normal distribution.

**Supplementary Table S4: Identified Outliers for ACS Prediction**
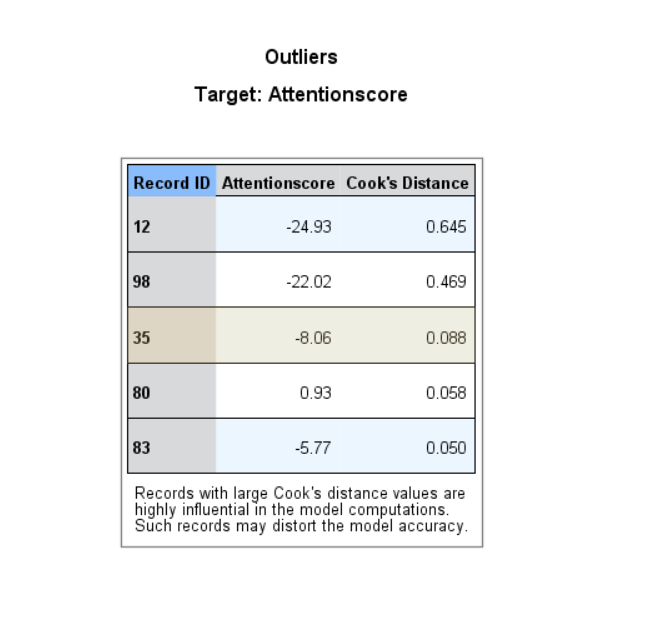


The table presents records with high Cook’s distance values, indicating their potential influence on the Attention Control Score (ACS) prediction model. Records with larger Cook’s distance values are more likely to distort the model's accuracy and should be carefully evaluated.

**Supplementary Table S5: Effects of Predictors on ACS Prediction**


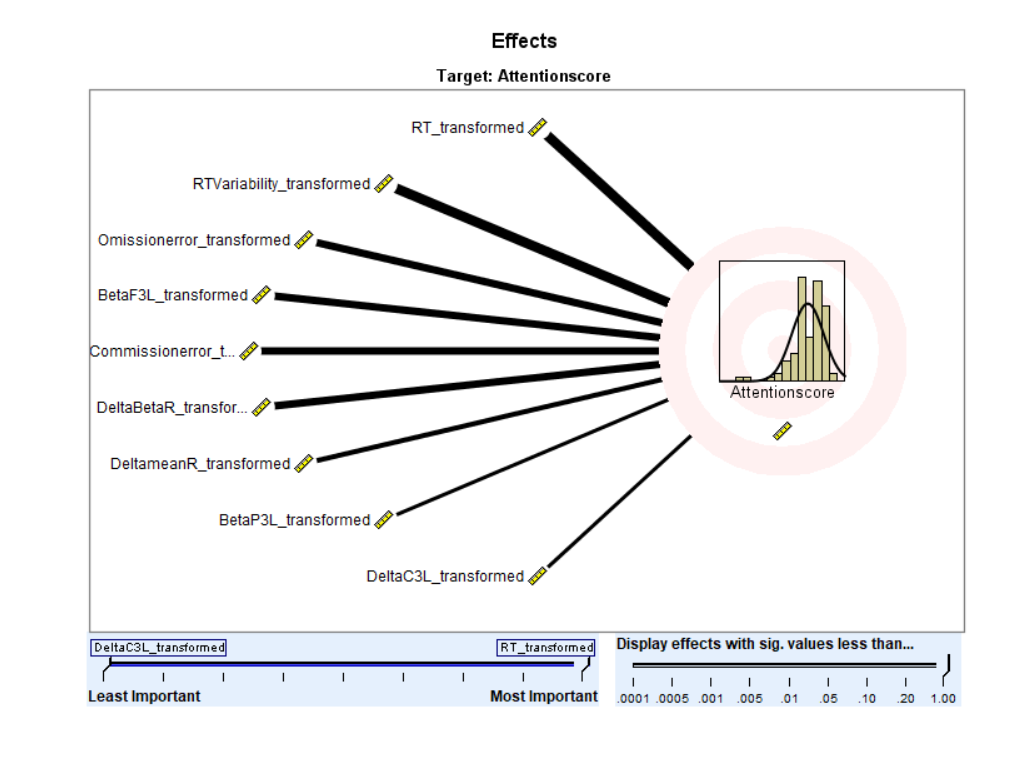


This diagram illustrates the relative effects of transformed predictors on Attention Control Score (ACS) prediction. The thickness of the lines indicates the strength of the relationship between each predictor and the ACS. Variables with thicker lines have a stronger influence on the model's outcome.

**Supplementary Table S6: Model Building Summary for ACS Prediction**


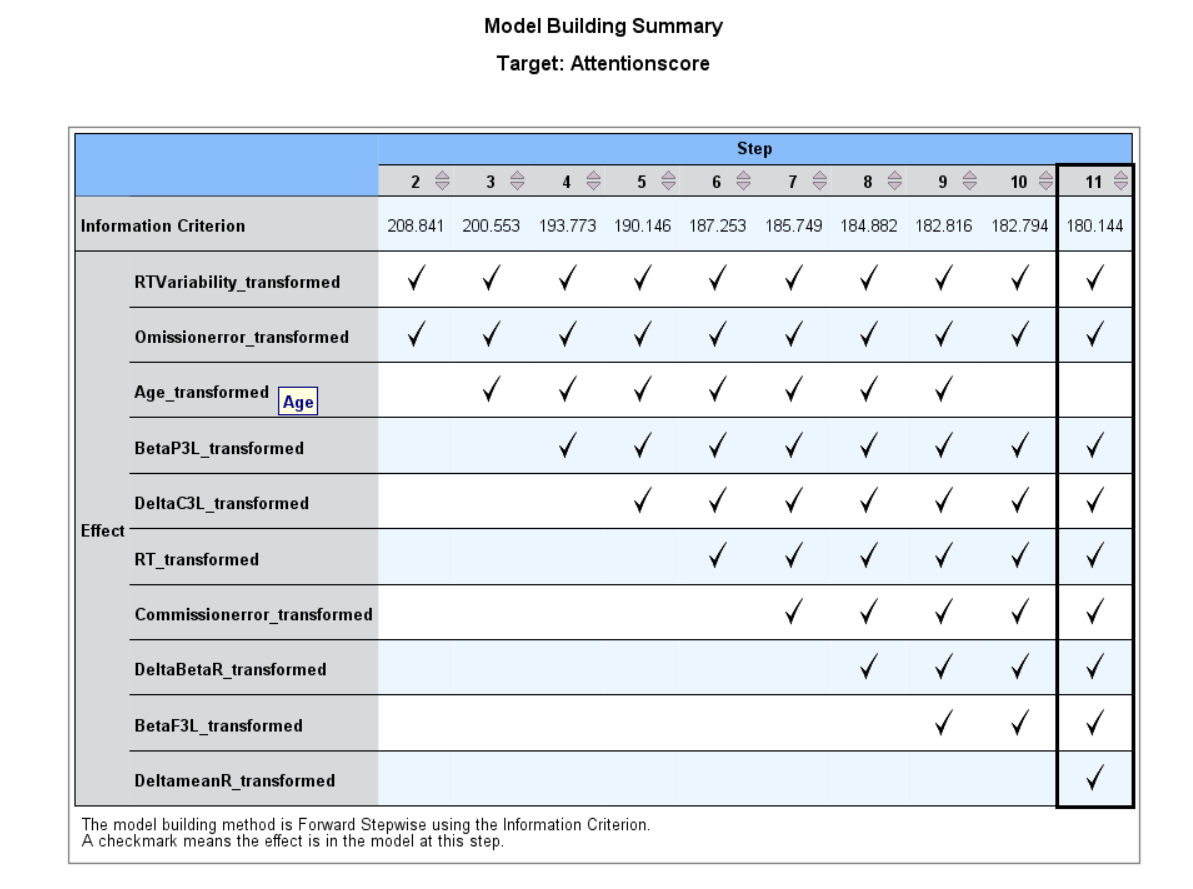


This table summarizes the forward stepwise model-building process for predicting the Attention Control Score (ACS) using the Information Criterion. A checkmark indicates that the corresponding predictor was included in the model at that step. The final model (Step 11) represents the most optimized combination of predictors.

**RTV Prediction**

**Supplementary Table S7: Predictor Importance for RTV Variability Prediction**


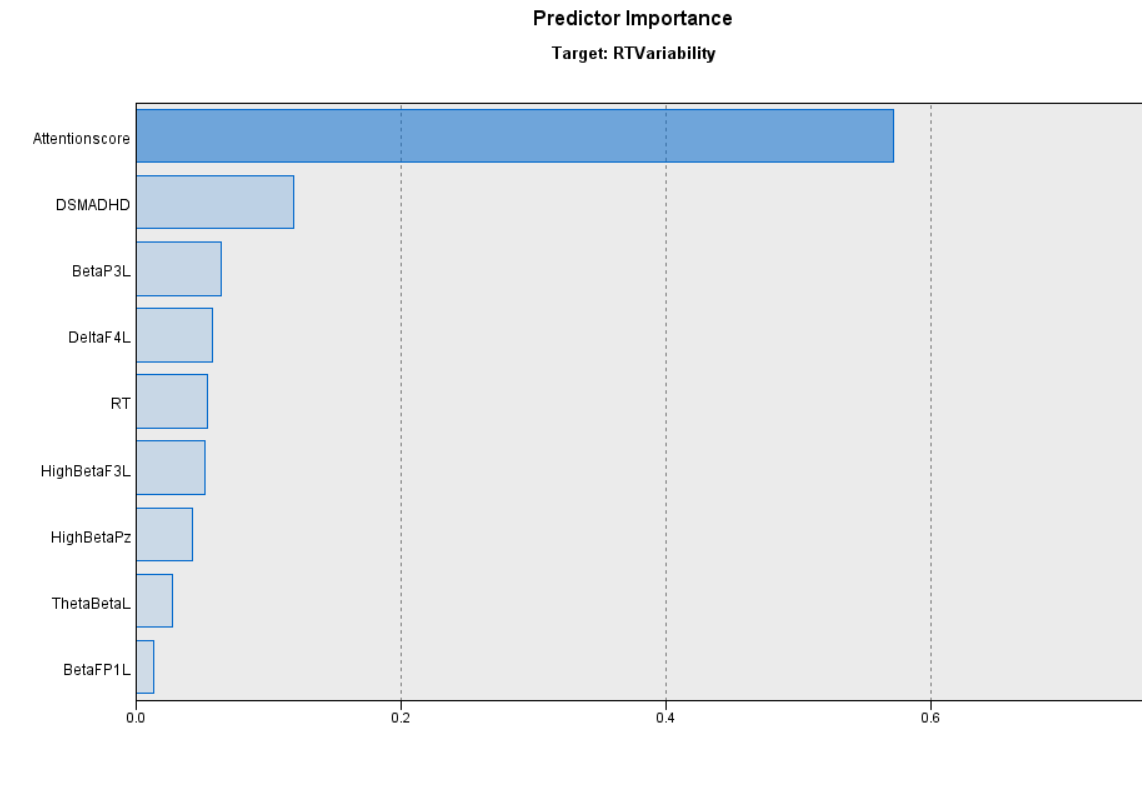


This chart illustrates the relative importance of predictors in the model for predicting RTV (Reaction Time Variability). Higher values indicate greater contribution of the respective predictor to the model. Attention Score emerged as the most significant predictor for RTV Variability.

**Supplementary Table S8: Residual Distribution for RTV Variability Prediction**


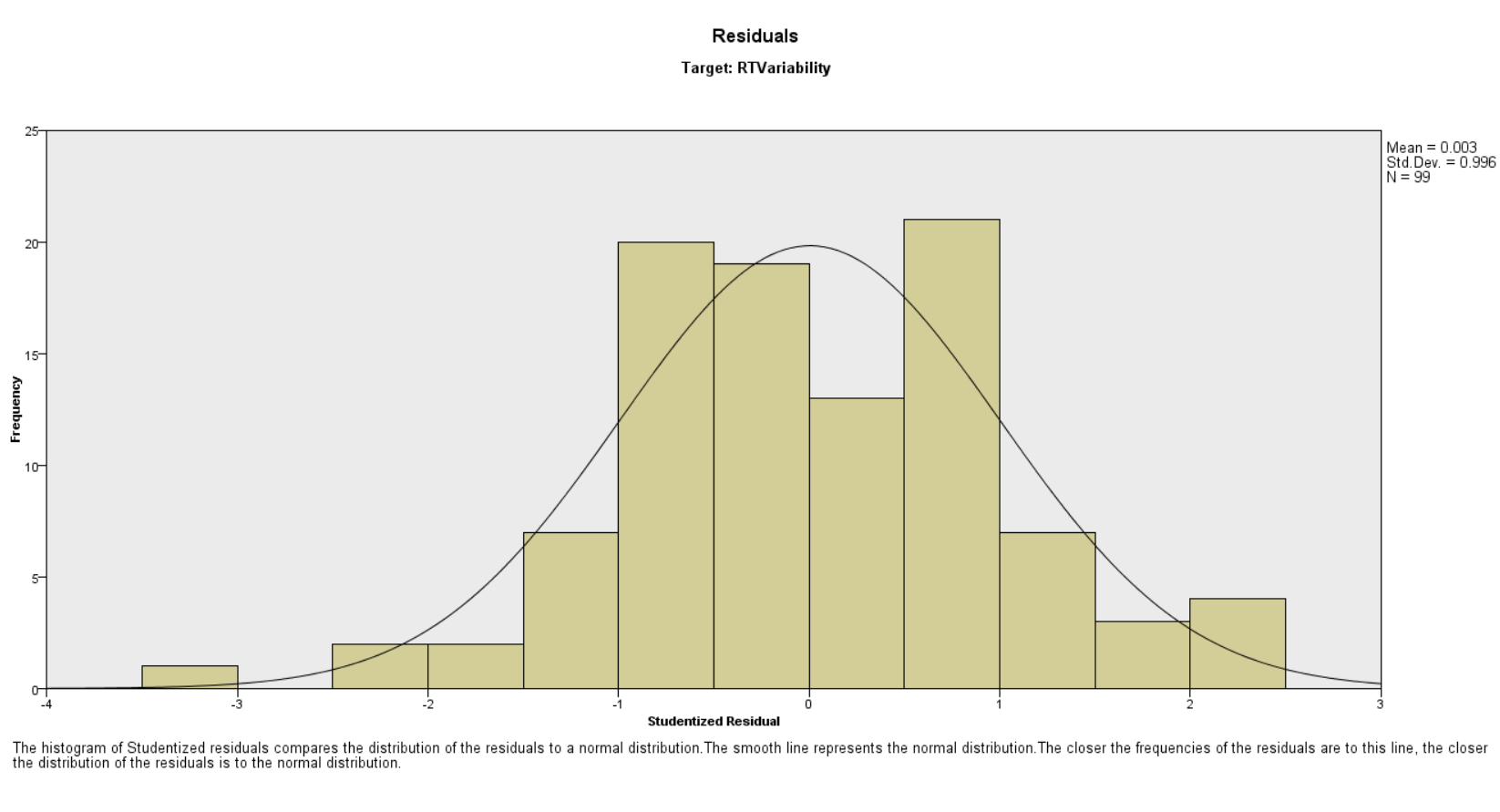


**The histogram displays the distribution of studentized residuals for the RTV (Reaction Time Variability) prediction model. The smooth line represents the normal distribution. The alignment of residual frequencies with the curve indicates the goodness-of-fit of the model.**

**Supplemantary Table S9: Identified Outliers for RTV Variability Prediction**


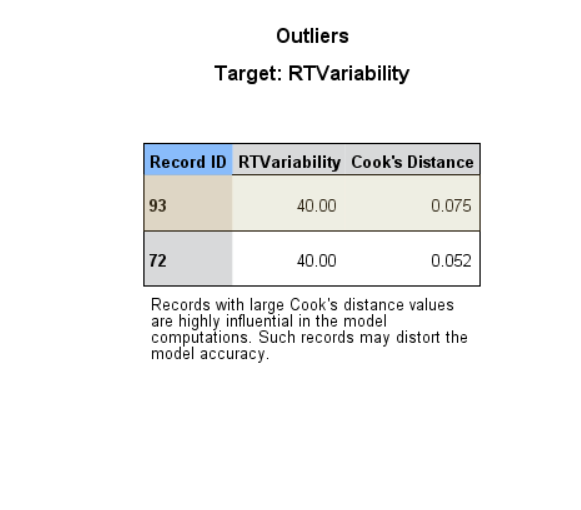


This table lists the records identified as outliers in the RTV (Reaction Time Variability) prediction model based on Cook's distance values. Records with larger Cook’s distance values indicate higher influence on the model computations, potentially affecting the model's accuracy.

**Supplemantary Table S10: Effects of Predictors on RTV Variability Prediction**


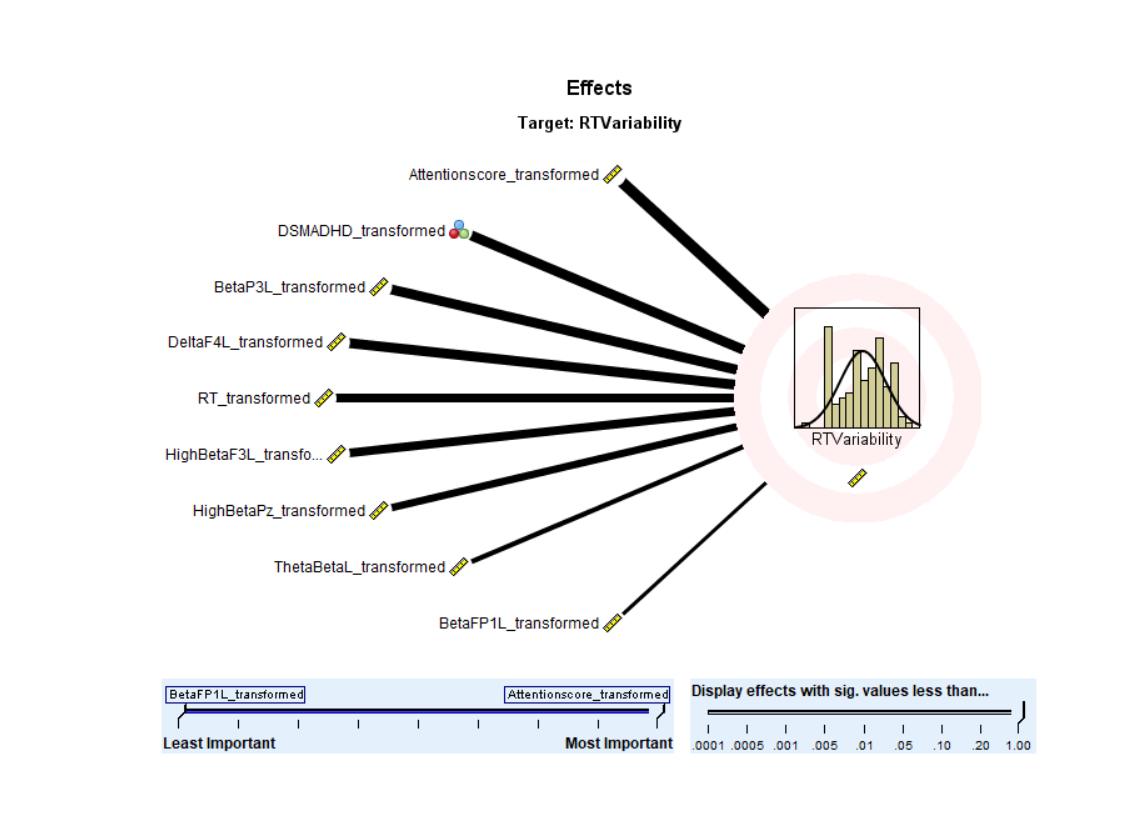


This diagram illustrates the effects of transformed predictors on the RTV (Reaction Time Variability) prediction model. The thickness of each line represents the relative influence of the corresponding predictor on RTV variability. Stronger effects are indicated by thicker lines, highlighting the most significant variables in the model.

**Supplemantary Table S11: Model Building Summary for RTV Variability Prediction**


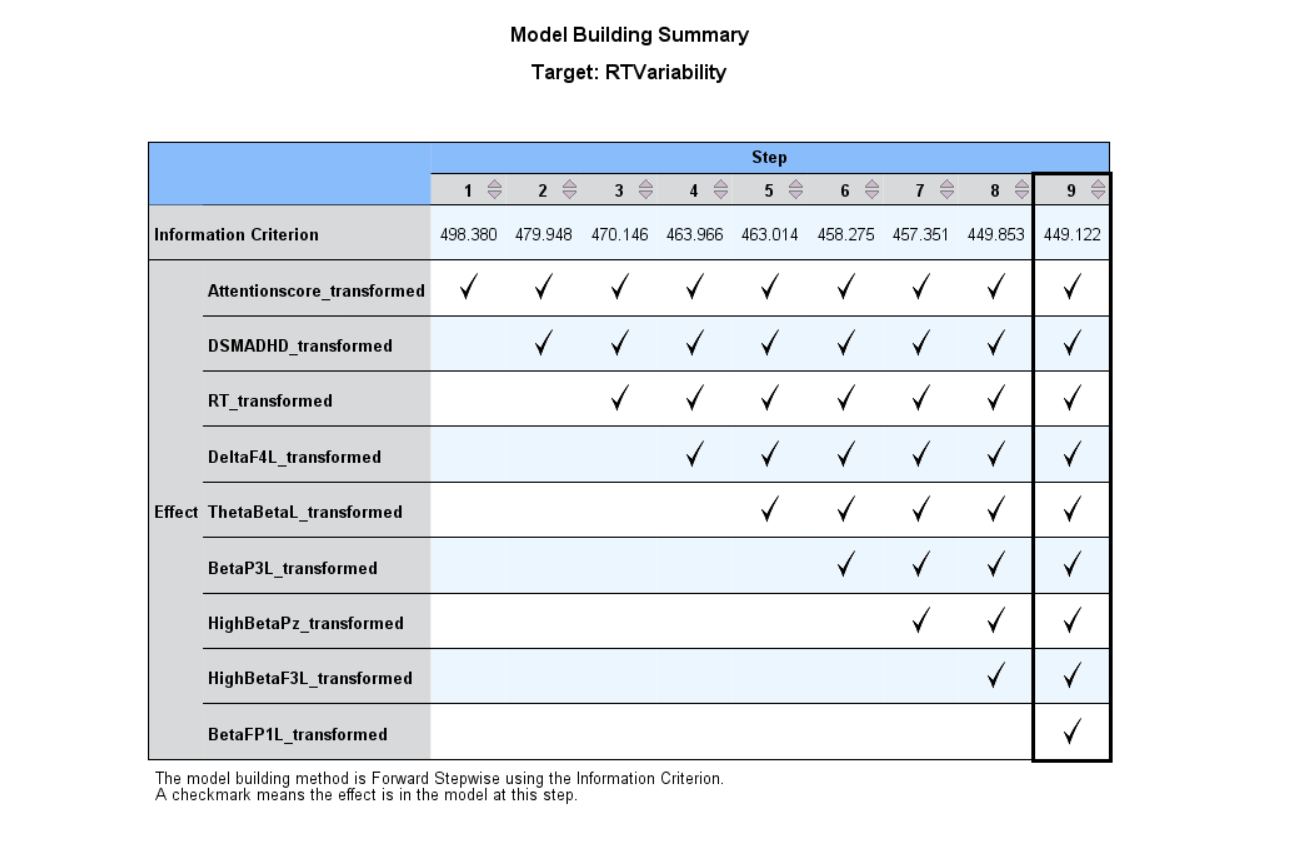


This table summarizes the forward stepwise model-building process for predicting RTV (Reaction Time Variability) using the Information Criterion. A checkmark indicates that the corresponding predictor was included in the model at that specific step. The final model (Step 9) represents the optimal combination of predictors for RTV variability.

**RT Prediction**

**Supplemantary Table S12: Predictor Importance for RT Prediction**

**
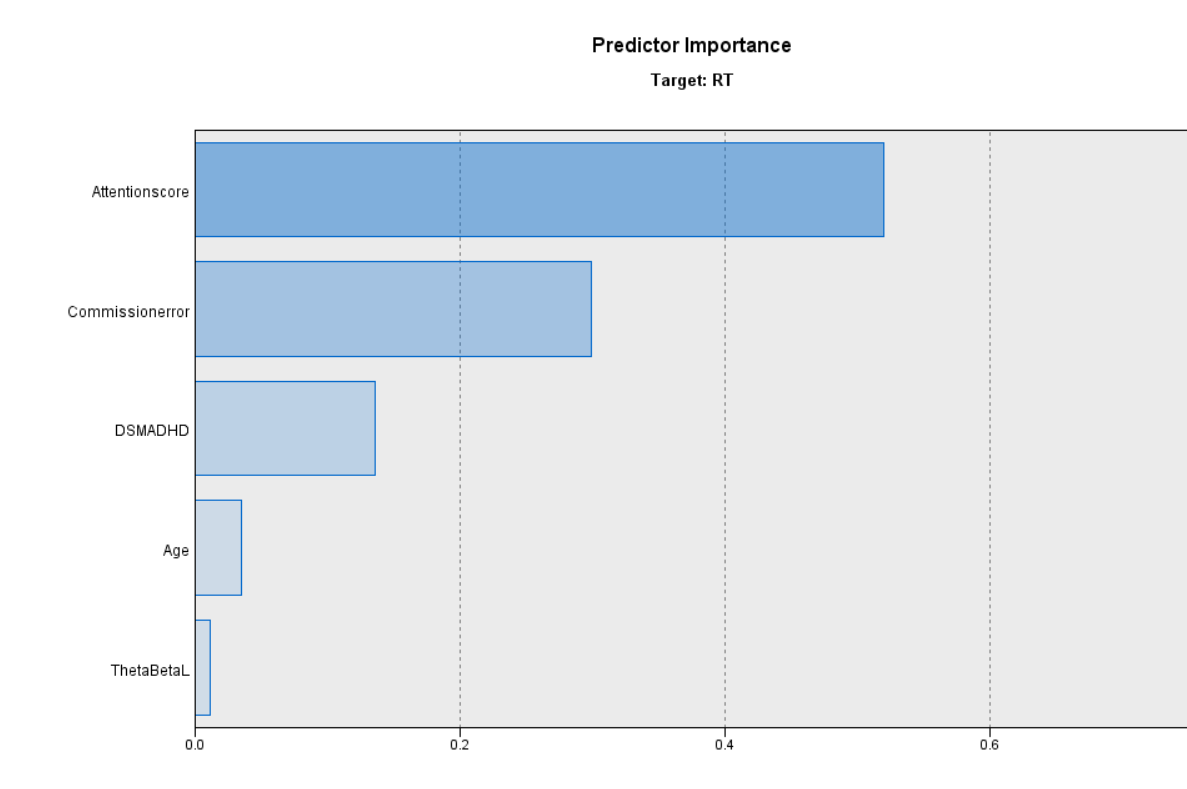
**

This chart illustrates the relative importance of predictors in the model for predicting RT (Reaction Time). Higher bars represent predictors with greater influence on the model. Attention Score and Commission Error were identified as the most significant predictors of RT.

**Supplemantary Table S13: Residual Distribution for RT Prediction**

**
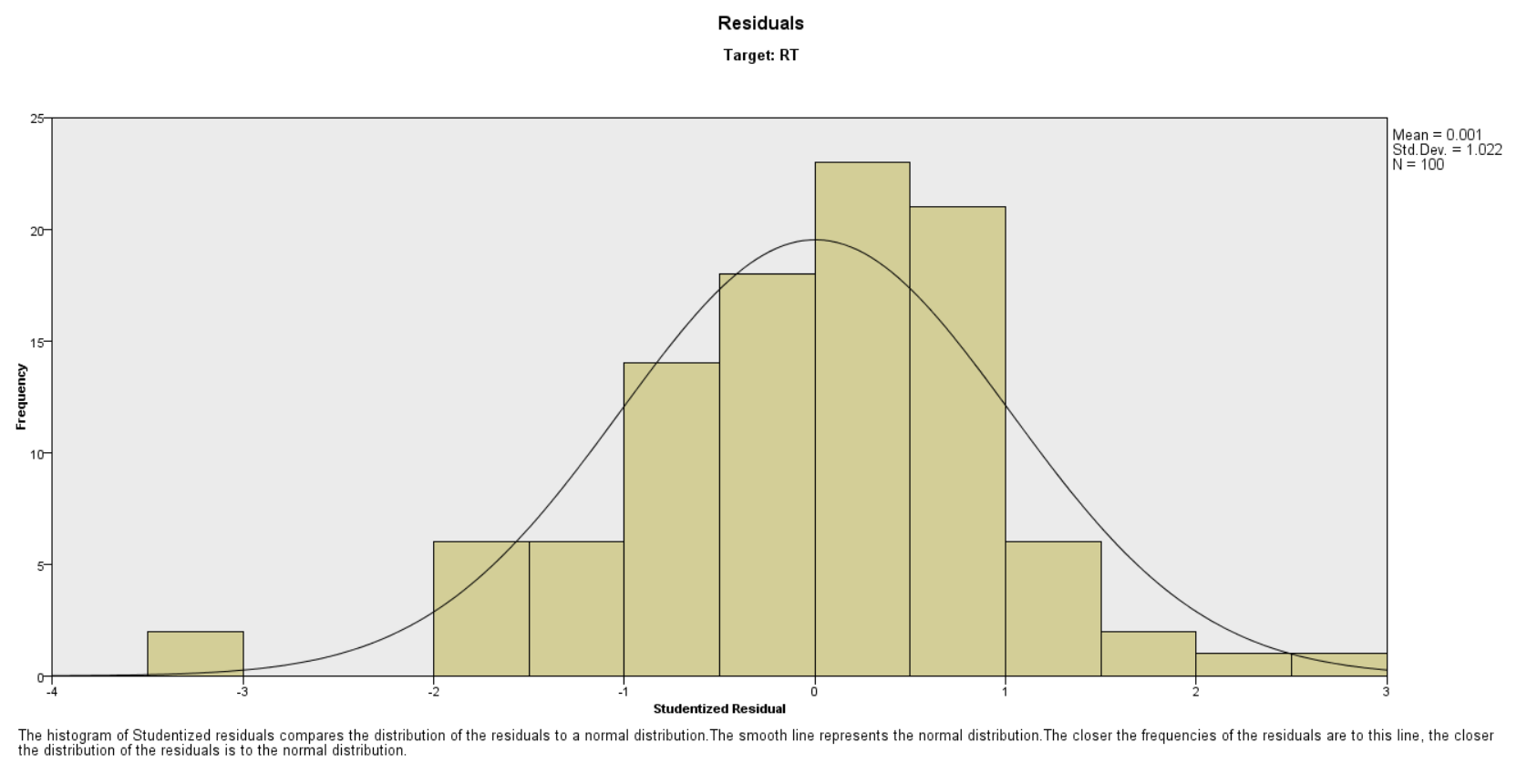
**

The histogram displays the distribution of studentized residuals for the Reaction Time (RT) prediction model. The smooth line represents the normal distribution. The alignment of residual frequencies with the curve indicates how well the residuals fit a normal distribution, reflecting the model's adequacy.

**Supplemantary** **Table S14: Identified Outliers for RT Prediction**

**
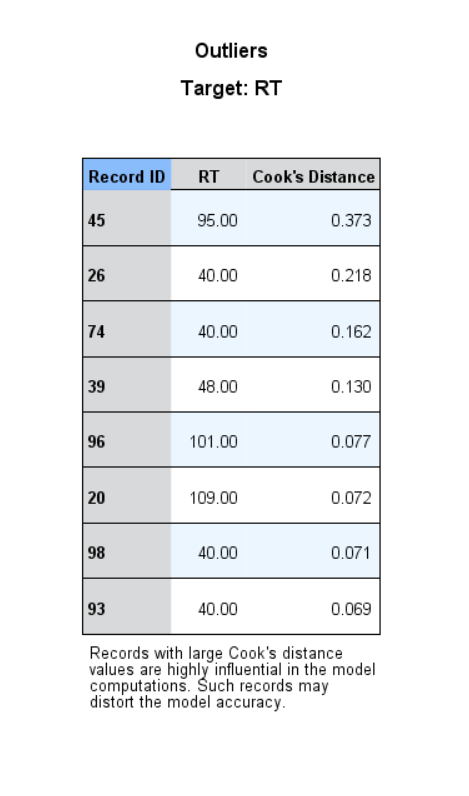
**

This table lists the records identified as outliers in the Reaction Time (RT) prediction model based on Cook's distance values. Records with higher Cook's distance values have a significant influence on the model and may impact its accuracy. These records should be carefully evaluated for their potential effects.

**Supplementary Table S15: Effects of Predictors on RT Prediction**

**
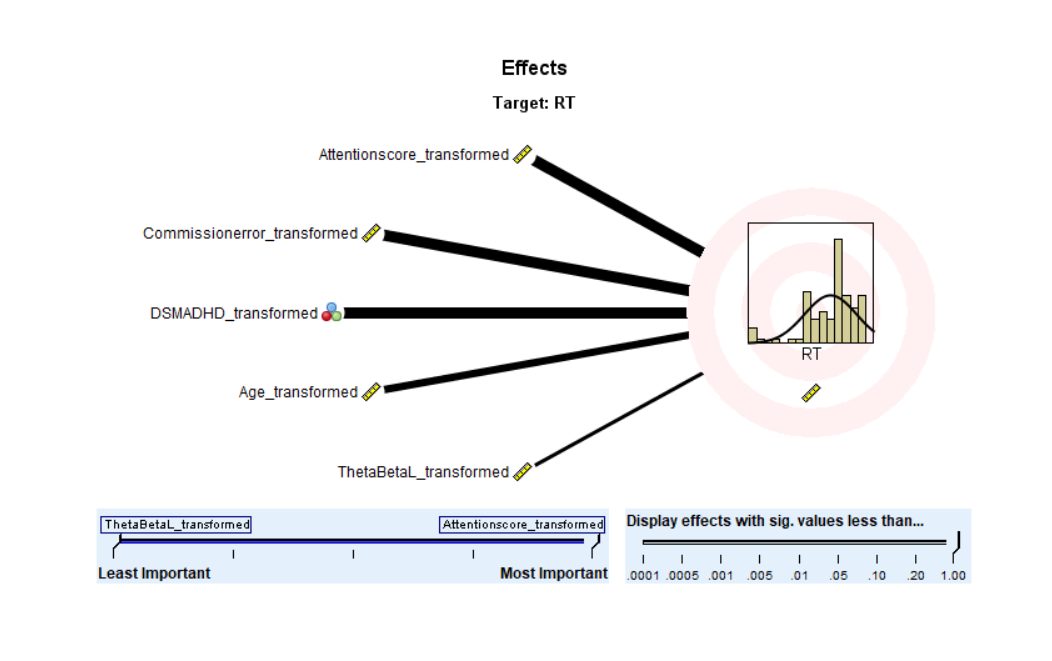
**

This diagram illustrates the effects of transformed predictors on the Reaction Time (RT) prediction model. The thickness of the lines represents the relative importance of each predictor, with thicker lines indicating stronger effects. Attention Score and Commission Error are shown to have the most significant influence on RT.

**Supplementary Table S16: Model Building Summary for RT Prediction**

**
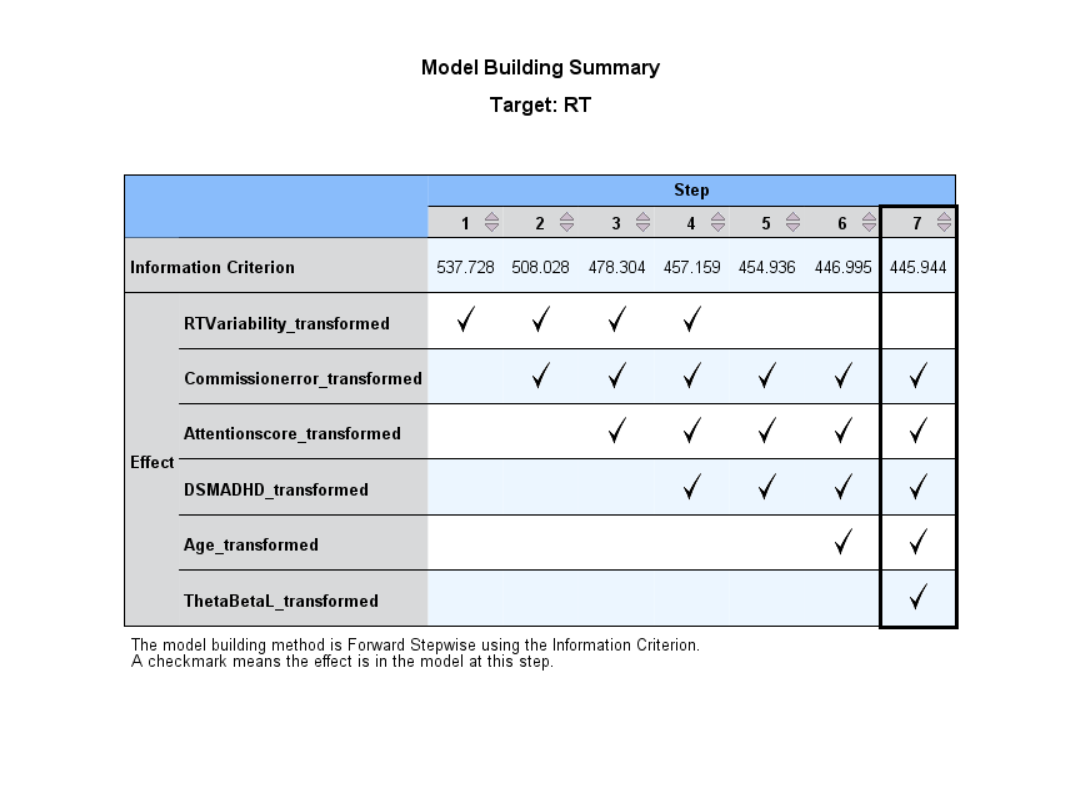
**

This table summarizes the forward stepwise model-building process for Reaction Time (RT) prediction using the Information Criterion. A checkmark indicates that the corresponding predictor was included in the model at the specified step. The final model (Step 7) represents the optimal set of predictors for RT prediction.

**Commission Error**

**Supplementary Table S17: Predictor Importance for Commission Error Prediction**

**
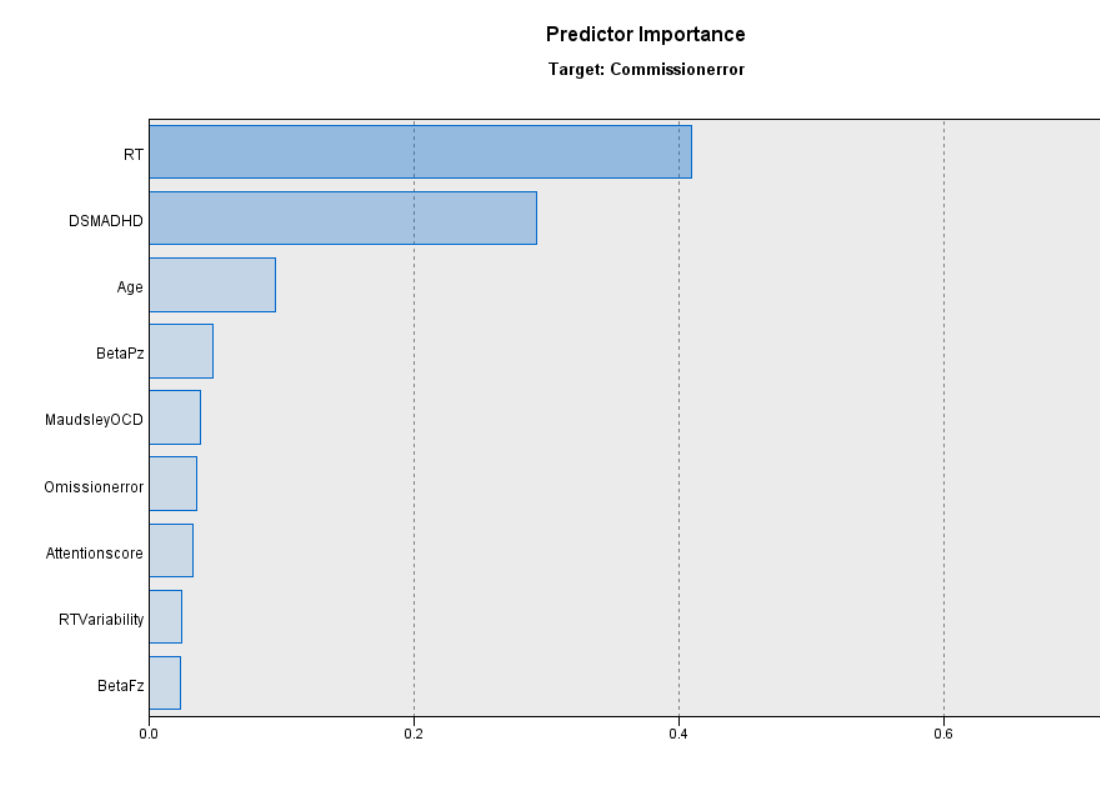
**

This chart illustrates the relative importance of predictors in the model for predicting Commission Error. RT (Reaction Time) and DSM-ADHD emerged as the most significant predictors, while other variables showed relatively smaller contributions to the model.

**Supplementary Table S18: Residual Distribution for Commission Error Prediction**

**
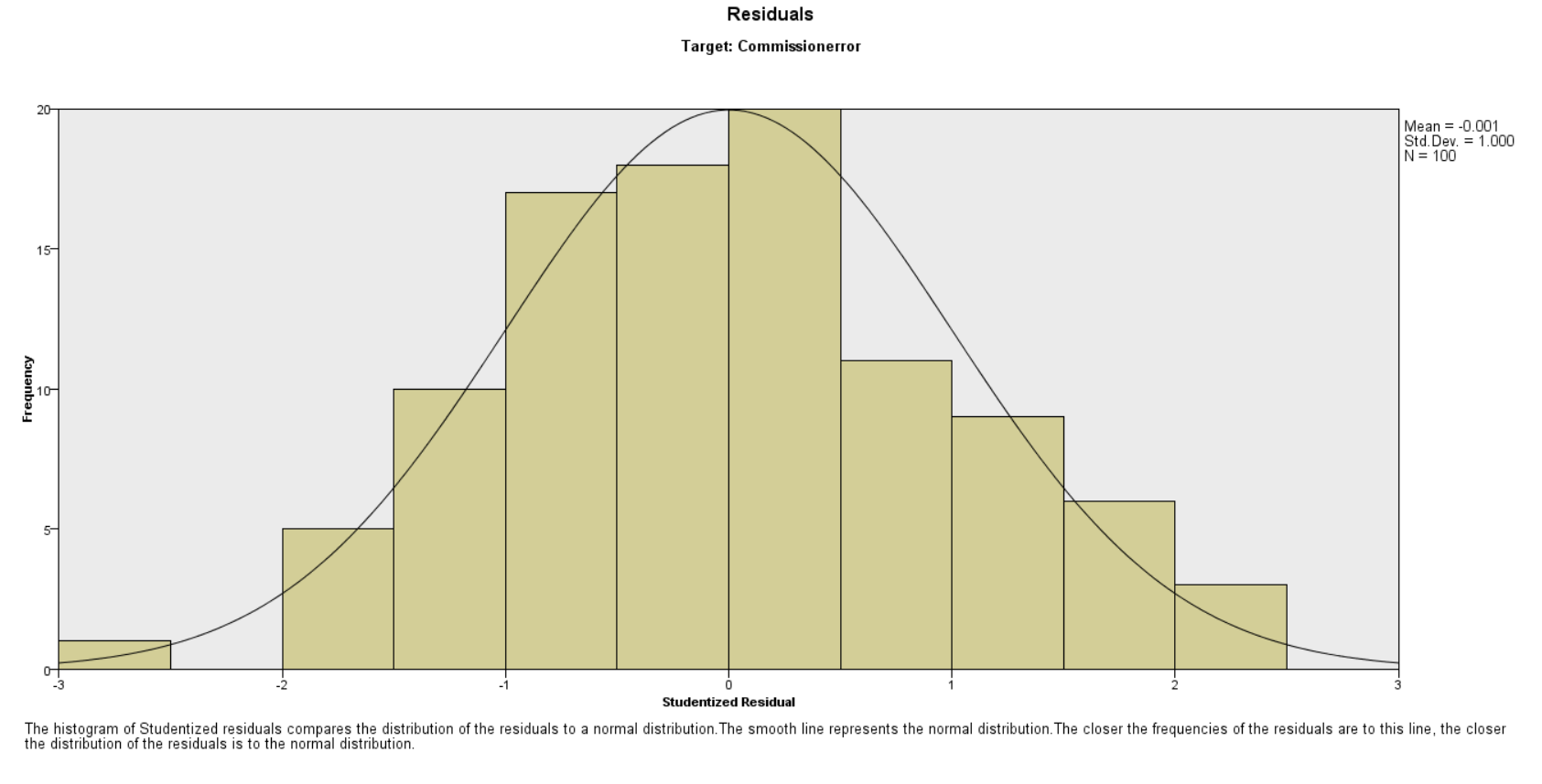
**

The histogram displays the distribution of studentized residuals for the Commission Error prediction model. The smooth line represents the normal distribution. The alignment of residual frequencies with the curve indicates the goodness-of-fit of the model and the adequacy of the residuals.

**Supplementary Table S19: Identified Outliers for Commission Error Prediction**

**
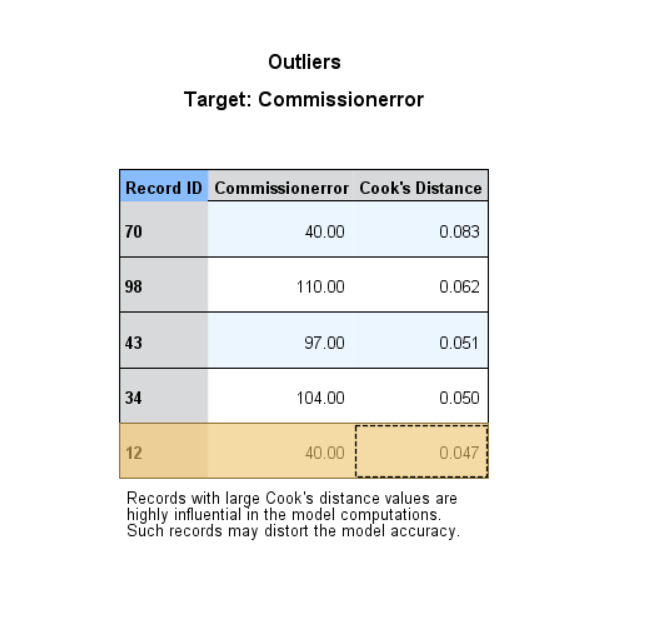
**

This table lists the records identified as outliers in the Commission Error prediction model based on Cook's distance values. Records with higher Cook's distance values significantly influence the model and may impact its accuracy. These records should be carefully evaluated for their potential effects on the results.

**Supplementary Table S20: Effects of Predictors on Commission Error Prediction**

**
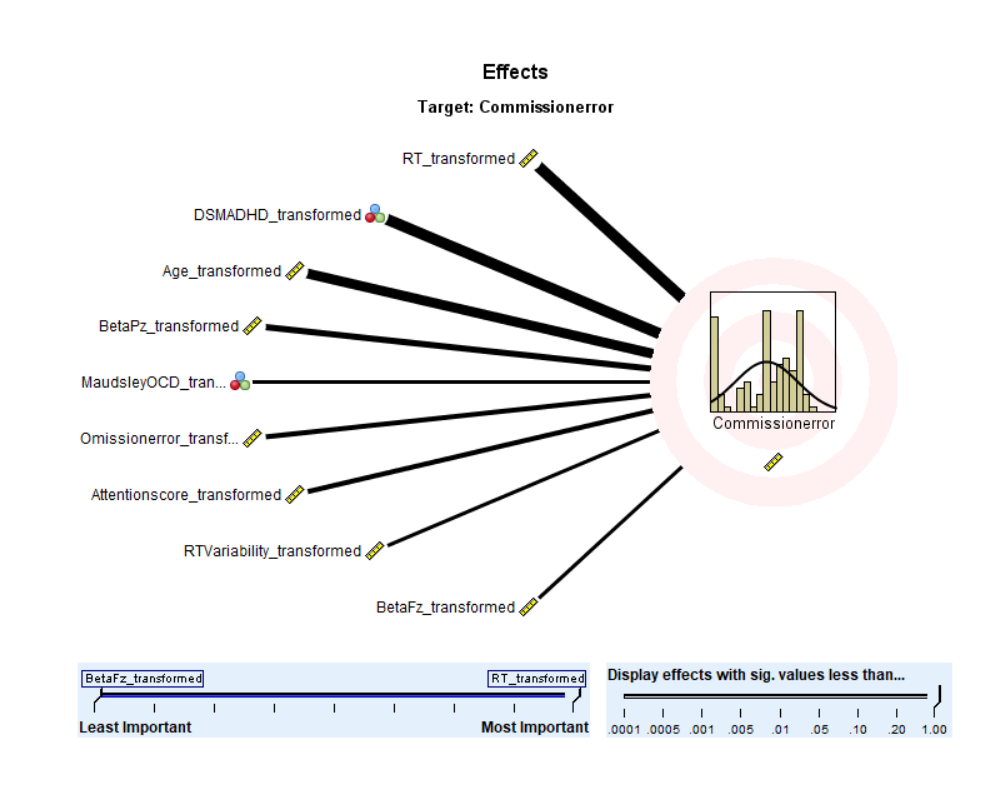
**

This diagram illustrates the effects of transformed predictors on the Commission Error prediction model. The thickness of the lines indicates the relative importance of each predictor, with RT (Reaction Time) showing the strongest effect. Other predictors, such as DSM-ADHD and Age, also contributed to the model with varying levels of influence.

**Supplementary** **Table S21: Model Building Summary for Commission Error Prediction**

**
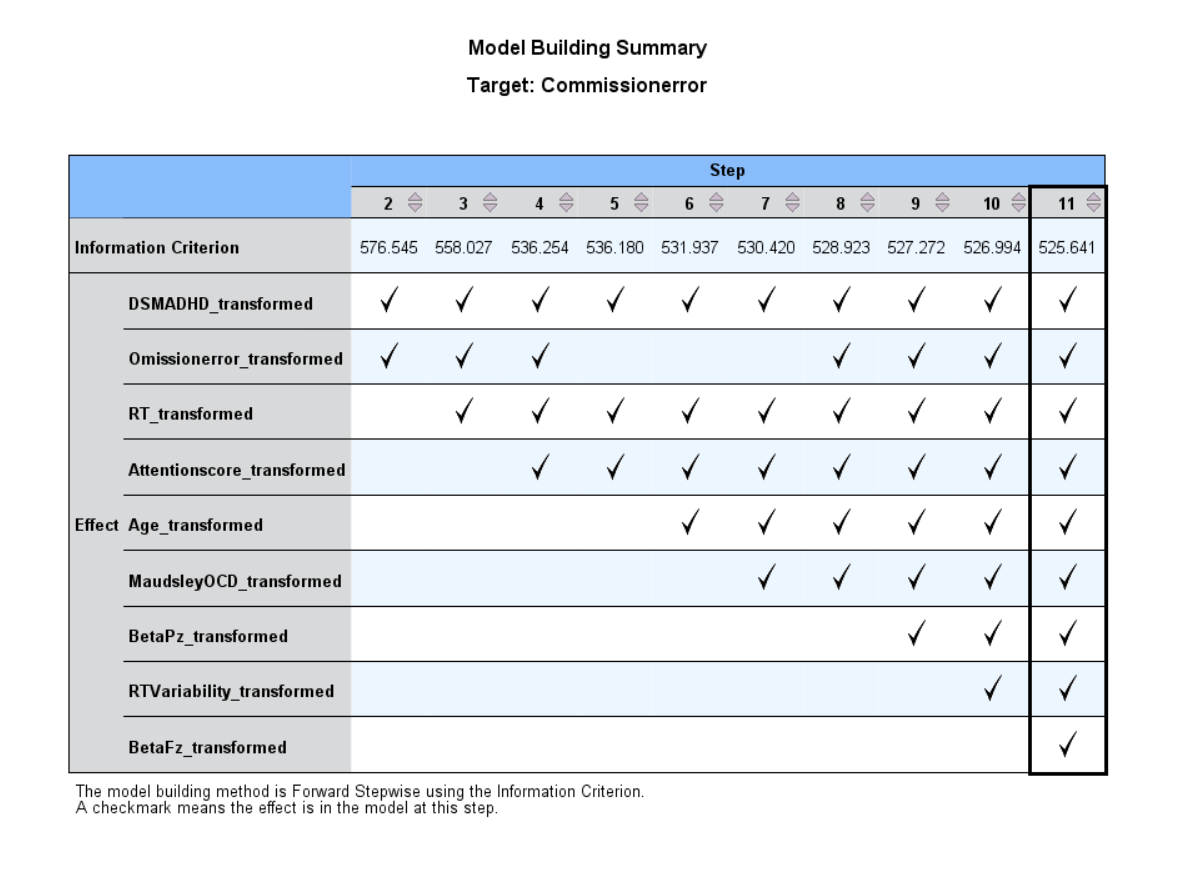
**

This table summarizes the forward stepwise model-building process for predicting Commission Error using the Information Criterion. A checkmark indicates that the corresponding predictor was included in the model at the specific step. The final model (Step 11) represents the optimal set of predictors contributing to Commission Error prediction.

**Omission Score Prediction**

**Supplementary Table S22: Predictor Importance for Omission Error Prediction**

**
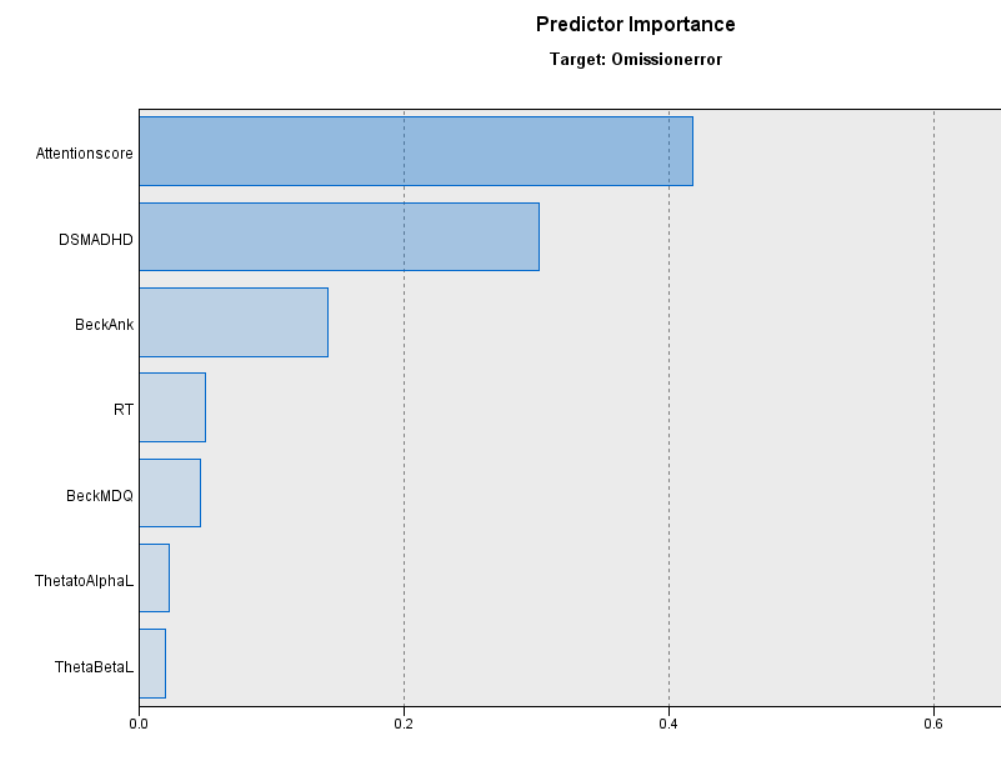
**

This chart illustrates the relative importance of predictors in the model for predicting Omission Errors. Attention Score emerged as the most significant predictor, followed by DSM-ADHD and Beck Anxiety scores. Other predictors showed smaller contributions to the model.

**Supplementary Table S23: Residual Distribution for Omission Error Prediction**

**
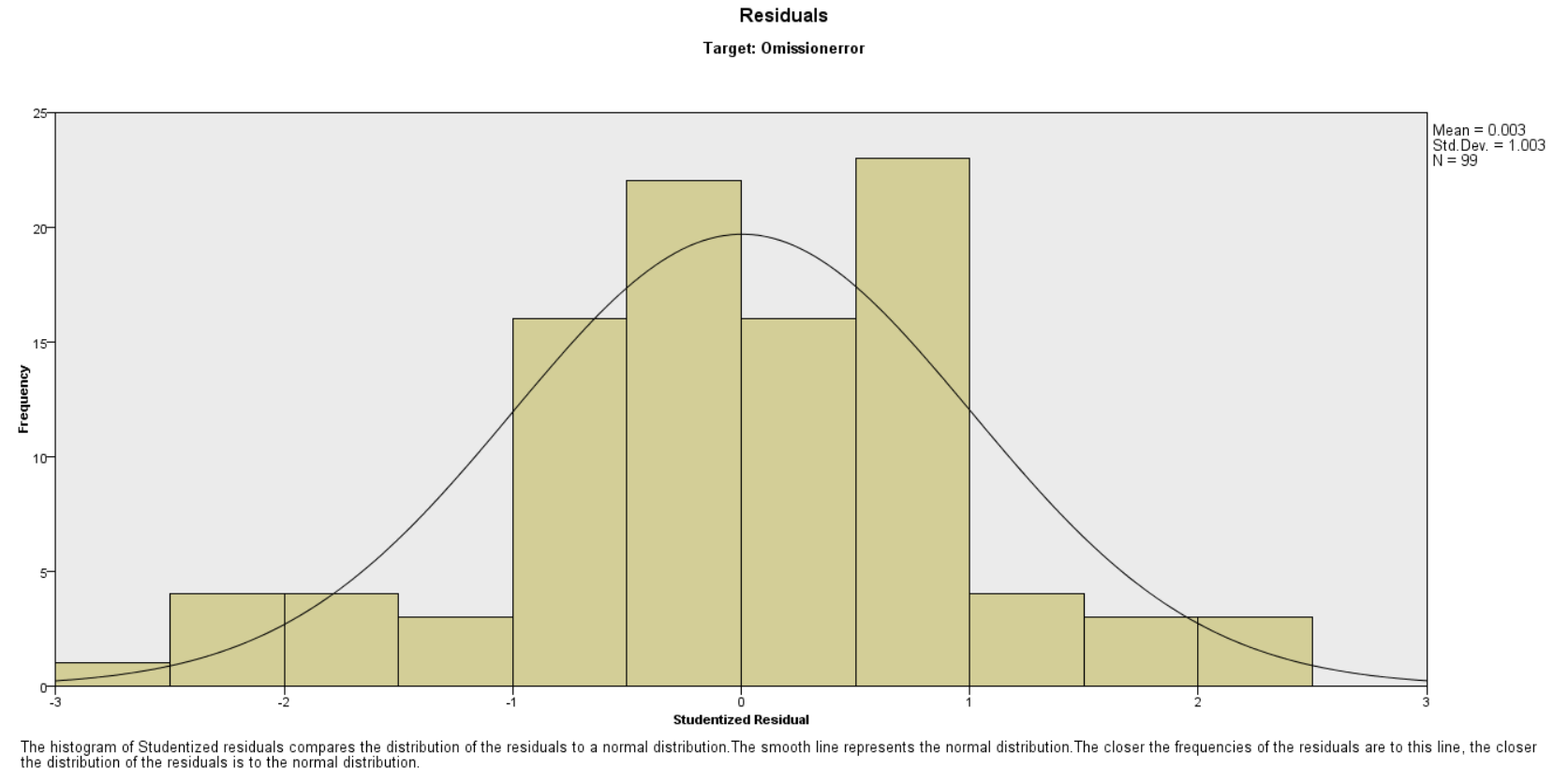
**

The histogram displays the distribution of studentized residuals for the Omission Error prediction model. The smooth line represents the normal distribution. The closer the residual frequencies align with the curve, the better the residuals fit a normal distribution, indicating the adequacy of the model.

**Supplementary Table S24: Identified Outliers for Omission Error Prediction**

**
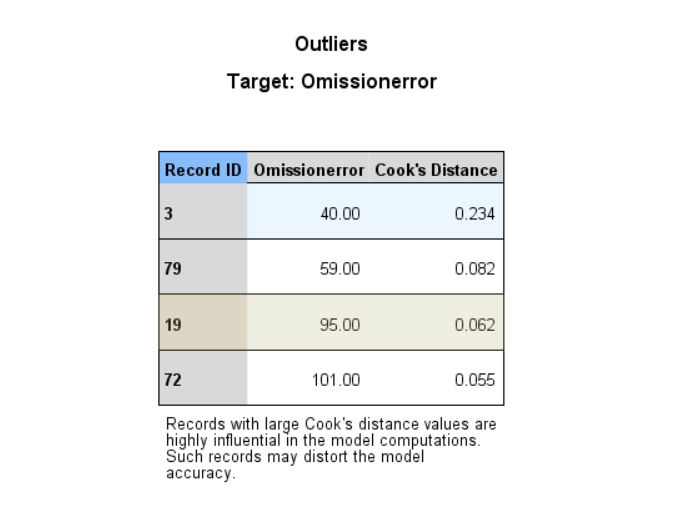
**

This table lists the records identified as outliers in the Omission Error prediction model based on Cook's distance values. Records with larger Cook's distance values indicate greater influence on the model computations and may impact the accuracy of the results. These records should be carefully evaluated for their effect on the model.

**Supplementary Table S25: Effects of Predictors on Omission Error Prediction**

**
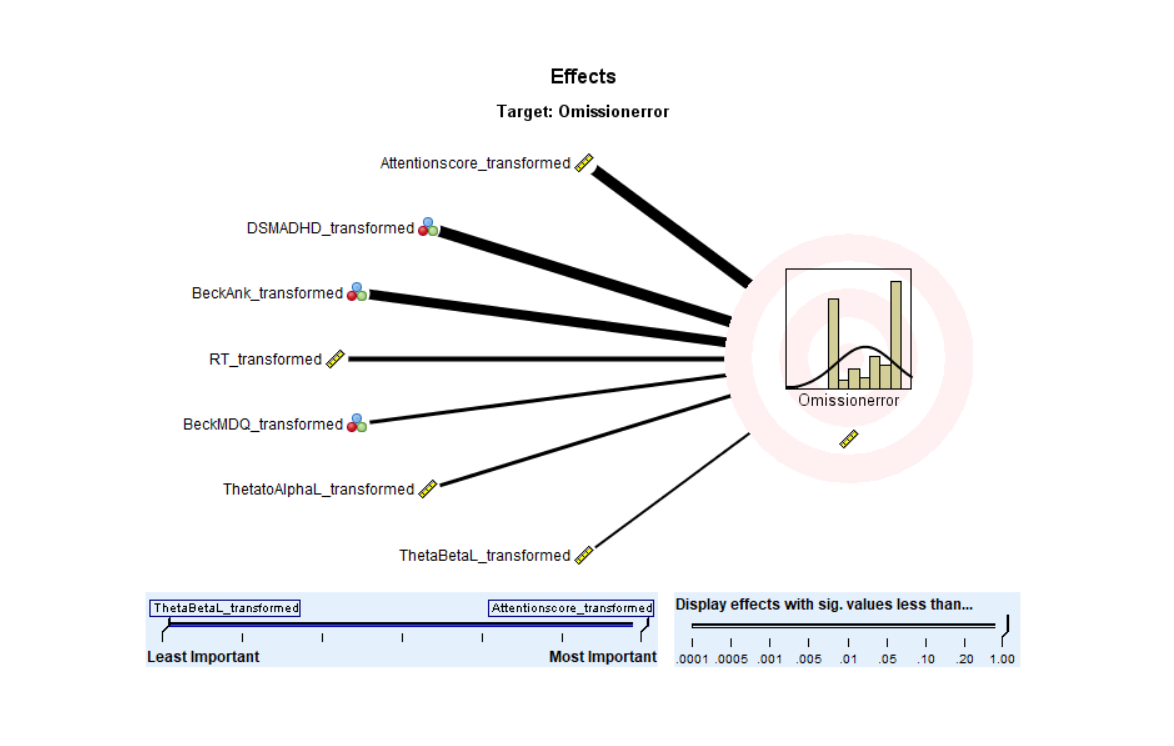
**

This diagram illustrates the effects of transformed predictors on the Omission Error prediction model. The thickness of each line represents the relative importance of the predictor, with Attention Score and DSM-ADHD showing the strongest effects. Other predictors, such as Beck Anxiety and Reaction Time, also contributed to the model with varying degrees of influence.

**Supplementary** **Table S26: Model Building Summary for Omission Error Prediction**

**
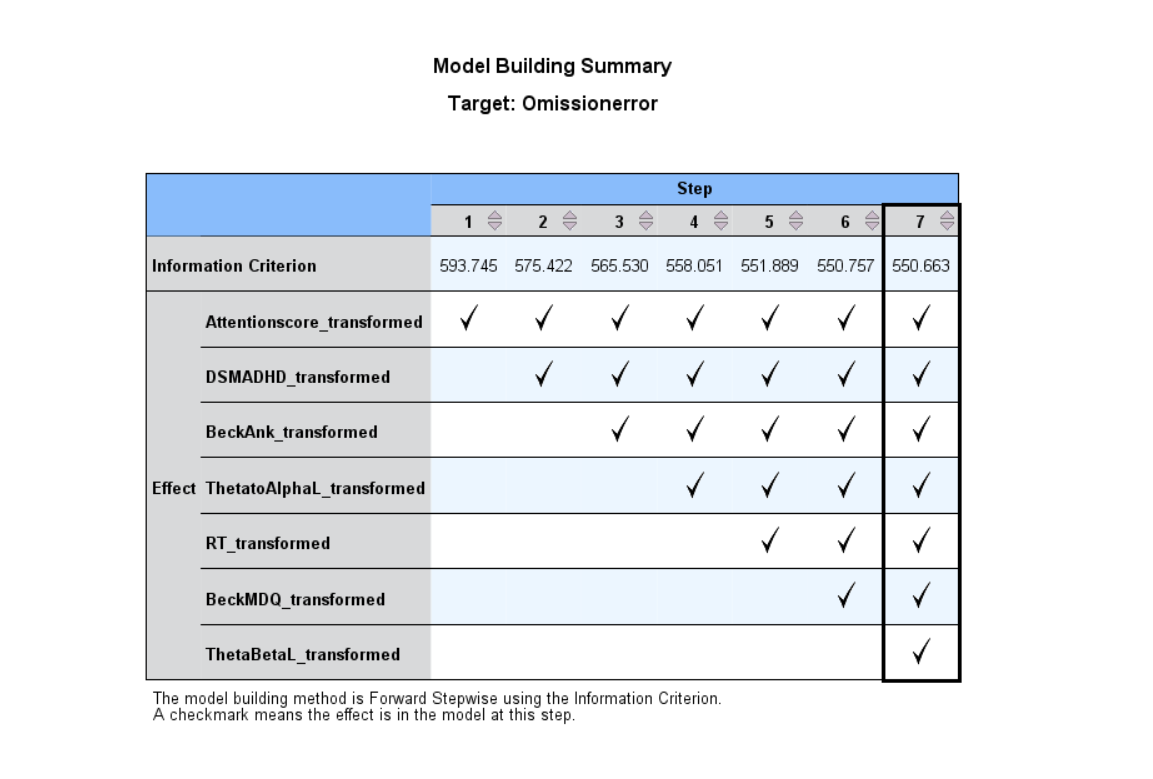
**

This table summarizes the forward stepwise model-building process for predicting Omission Errors using the Information Criterion. A checkmark indicates that the corresponding predictor was included in the model at that specific step. The final model (Step 7) represents the optimal set of predictors for Omission Error prediction.
